# Converging mechanism of UM171 and KBTBD4 neomorphic cancer mutations

**DOI:** 10.1038/s41586-024-08533-3

**Published:** 2025-02-12

**Authors:** Xiaowen Xie, Olivia Zhang, Megan J. R. Yeo, Ceejay Lee, Ran Tao, Stefan A. Harry, N. Connor Payne, Eunju Nam, Leena Paul, Yiran Li, Hui Si Kwok, Hanjie Jiang, Haibin Mao, Jennifer L. Hadley, Hong Lin, Melissa Batts, Pallavi M. Gosavi, Vincenzo D’Angiolella, Philip A. Cole, Ralph Mazitschek, Paul A. Northcott, Ning Zheng, Brian B. Liau

**Affiliations:** 1 Department of Pharmacology, University of Washington, Seattle, WA USA; 2 Howard Hughes Medical Institute, University of Washington, Seattle, WA USA; 3 Department of Chemistry and Chemical Biology, Harvard University, Cambridge, MA USA; 4 Broad Institute of MIT and Harvard, Cambridge, MA USA; 5 Center of Excellence in Neuro-Oncology Sciences, St. Jude Children’s Research Hospital, Memphis, TN USA; 6 Department of Developmental Neurobiology, St. Jude Children’s Research Hospital, Memphis, TN USA; 7 Center for Systems Biology, Massachusetts General Hospital, Boston, MA USA; 8 Harvard T.H. Chan School of Public Health, Boston, MA USA; 9 Division of Genetics, Department of Medicine, Brigham and Women’s Hospital, Department of Biological Chemistry and Molecular Pharmacology, Harvard Medical School, Boston, MA USA; 10 Edinburgh Cancer Research, Cancer Research UK Scotland Centre, The Institute of Genetics and Cancer, University of Edinburgh, Edinburgh, UK

**Keywords:** Paediatric cancer, Cryoelectron microscopy, Mutagenesis, Small molecules

## Abstract

Cancer mutations can create neomorphic protein–protein interactions to drive aberrant function^1,2^. As a substrate receptor of the CULLIN3-RING E3 ubiquitin ligase complex, KBTBD4 is recurrently mutated in medulloblastoma^3^, the most common embryonal brain tumour in children^4^. These mutations impart gain-of-function to KBTBD4 to induce aberrant degradation of the transcriptional corepressor CoREST^5^. However, their mechanism remains unresolved. Here we establish that KBTBD4 mutations promote CoREST degradation through engaging HDAC1/2 as the direct target of the mutant substrate receptor. Using deep mutational scanning, we chart the mutational landscape of the KBTBD4 cancer hotspot, revealing distinct preferences by which insertions and substitutions can promote gain-of-function and the critical residues involved in the hotspot interaction. Cryo-electron microscopy analysis of two distinct KBTBD4 cancer mutants bound to LSD1–HDAC1–CoREST reveals that a KBTBD4 homodimer asymmetrically engages HDAC1 with two KELCH-repeat β-propeller domains. The interface between HDAC1 and one of the KBTBD4 β-propellers is stabilized by the medulloblastoma mutations, which insert a bulky side chain into the HDAC1 active site pocket. Our structural and mutational analyses inform how this hotspot E3–neosubstrate interface can be chemically modulated. First, we unveil a converging shape-complementarity-based mechanism between gain-of-function E3 mutations and a molecular glue degrader, UM171. Second, we demonstrate that HDAC1/2 inhibitors can block the mutant KBTBD4–HDAC1 interface and proliferation of KBTBD4-mutant medulloblastoma cells. Altogether, our work reveals the structural and mechanistic basis of cancer mutation-driven neomorphic protein–protein interactions.

## Abstract (web summary)

We show that gain-of-function cancer mutations in the KBTBD4 E3 ligase promote neodegradation of substrates via a shape-complementarity-based mechanism, which converges with the mechanism of action of the UM171 molecular glue degrader and can be blocked by HDAC1/2 inhibitors.

## Main

Human genetic variation and somatic mutations in protein-coding genes can alter their protein–protein interactions (PPIs) to drive disease states^1,2,6^. Although many of these mutations cause loss-of-function, recent studies have demonstrated how they can also promote neomorphic PPIs with aberrant functions^2,7–9^. Understanding the molecular mechanisms governing how mutations can enable ‘neo-PPIs’ will be critical not only for understanding disease aetiology but also for guiding therapeutic modalities, such as molecular glues and PPI inhibitors, to chemically modulate these interfaces^10,11^.

In the ubiquitin–proteasome system, human disease mutations have long been known to compromise the functions of several E3 ubiquitin ligases^12–15^. By contrast, gain-of-function mutations in E3s promoting aberrant degradation of substrate proteins have only recently emerged as a fascinating phenomenon. Although cases of hypermorphic E3 mutations are documented, leading to unscheduled substrate ubiquitination by altering ligase stability or regulation^16–19^, neomorphic E3 mutations that directly induce neosubstrate engagement and degradation, ‘neodegradation’, represent a new paradigm in E3 ligase dysregulation.

**Fig. 1:**
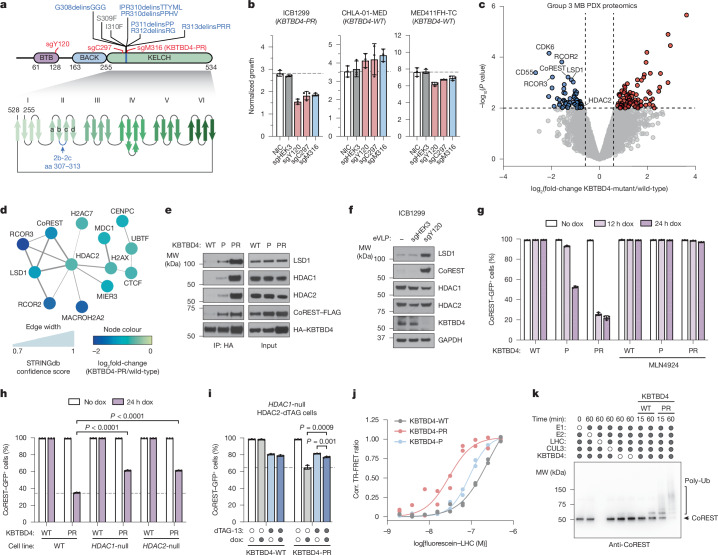
KBTBD4 MB mutants potentiate E3 activity. **a**, Schematic of KBTBD4 protein domains and recurrent MB mutations. **b**, Normalized ex vivo proliferation over 7 days for indicated MB models after transduction with eVLPs. **c**, Whole-proteome quantification in *KBTBD4*^*MUT*^ (*n* = 2) versus *KBTBD4*^*WT*^ (*n* = 5) PDX models. Coloured dots show proteins with |log_2_(fold-change)| > 0.7 in *KBTBD4*^*MUT*^ versus *KBTBD4*^*WT*^ and *P* value < 0.01 (empirical Bayes-moderated *t*-tests). **d**, STRING network of proteins significantly depleted in *KBTBD4*^*MUT*^ (*n* = 2) versus *KBTBD4*^*WT*^ (*n* = 5) PDX models. Edge width scale depicts PPI confidence. **e**, HA IP immunoblots from 293T cells transfected with FLAG–CoREST and indicated HA–KBTBD4 variants treated with MLN4924 (1 µM) for 4 h. **f**, Immunoblots in ICB1299 after transduction with eVLPs. **g**, Flow cytometry quantification of GFP^+^ cells for *KBTBD4*-null CoREST–GFP cells after 1 h MLN4924 pre-treatment followed by dox-inducible overexpression of indicated KBTBD4 variant. **h**, Flow cytometry quantification of GFP^+^ cells for indicated CoREST–GFP cells with or without 24 h dox-inducible overexpression of KBTBD4-PR or KBTBD4-WT. **i**, Flow cytometry quantification of GFP^+^ cells for indicated CoREST–GFP cells with or without 24 h dox-inducible overexpression of KBTBD4-PR or KBTBD4-WT. Cells were pretreated for 2 h with DMSO or dTAG-13 (500 nM). **j**, TR-FRET signal between fluorescein–LHC and anti-His CoraFluor-1-labelled antibody with indicated His–KBTBD4 variant (*n* = 2 biological replicates). **k**, Immunoblots of in vitro ubiquitination assays of CRL3^KBTBD4-WT^ and CRL3^KBTBD4-PR^ with LHC (*n* = 3 biological replicates). Data in **b** and **g**–**i** are mean ± s.d. of *n* = 3 biological replicates and representative of two independent experiments. *P* values in **h** and **i** were calculated through two-tailed unpaired *t*-tests for indicated comparisons. Data in **e**,**f** and **j** are representative of two independent experiments. FACS-gating schemes and uncropped blots are shown in Supplementary Figs. 1a and 2, respectively. Corr., corrected; DMSO, dimethylsulfoxide; HA, haemagglutinin; IP, immunoprecipitation; MW, molecular weight; NIC, non-infection control. Source data

Cancer mutations in *KBTBD4*, a CULLIN3-RING E3 ligase (CRL3) substrate receptor, present the first compelling case of E3 ligase neomorphic mutations. *KBTBD4* is recurrently mutated in group 3 and 4 medulloblastomas (MBs)^3^, molecular subtypes associated with poor outcomes and lacking effective treatment options, as well as in pineal parenchymal tumours^4^. These mutations occur in a hotspot in the 2b-2c loop of the KELCH-repeat β-propeller and comprise considerable molecular diversity, spanning 1–5-amino-acid insertion–deletions (indels or delins) as well as point substitutions^3^ (Fig. 1a). The most common mutations include P311delinsPP and R313delinsPRR (abbreviated as P and PR, hereafter), which promote the neomorphic degradation of the CoREST and LSD1 subunits of the LSD1–HDAC1/2–CoREST (LHC) complex^5^. Notably, KBTBD4 is also involved in the mechanism of UM171, a small molecule agonist of haematopoietic stem cell expansion that induces CoREST degradation^20,21^. Despite these connections, the molecular target and mechanism of mutant KBTBD4 remain unclear. In conjunction with a companion study, here we reveal a striking mechanistic mimicry between UM171 and the *KBTBD4* cancer mutations.

## KBTBD4-PR promotes MB cell proliferation

**Extended Data Fig. 1:**
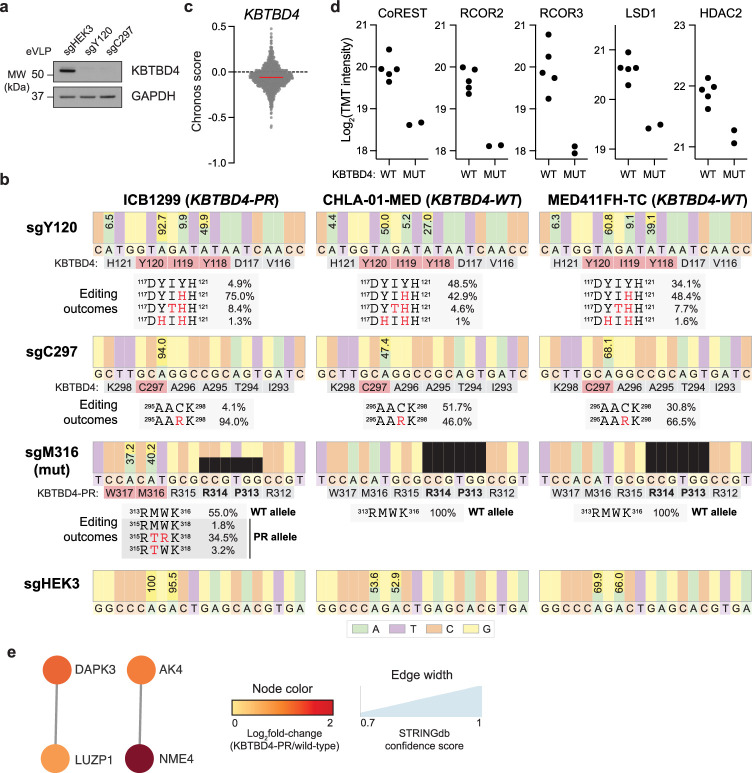
Supporting data for experiments involving PDX models. **a**, Immunoblots showing KBTBD4 and GAPDH in K562 cells after transduction of the indicated eVLPs. **b**, Base editing efficiency and mutation outcomes for ICB1299 (left), CHLA-01-MED (center), and MED411FH-TC (right). Numbers in bars indicate percentage of A-to-G edits, and black bars indicate the lack of inserted bases in *KBTBD4-WT*. Base editing outcomes for protein variants ≥1% frequency shown directly below. Amino acid mutations are shown in red. Genotyping was performed once. **c**, Scaled gene-dependency score (Chronos) of *KBTBD4* across all cell lines in the 24Q4 release of DepMap (gray dots, *n* = 1,150). Red bar indicates median value. **d**, Relative protein abundances (log_2_(tandem-mass tag (TMT) intensity)) in *KBTBD4*^*WT*^ (*n* = 5) and *KBTBD4*^*MUT*^ (*n* = 2) PDX models for selected proteins. **e**, STRING network of proteins enriched in *KBTBD4*^*MUT*^ (*n* = 2) versus WT (*n* = 5) PDX models. Node color scale depicts log_2_(fold-change) protein abundance in mutant versus WT models. Edge width scale depicts confidence of the PPI for the nodes. Immunoblots in **a** are representative of two independent experiments. Uncropped blots can be found in Supplementary Fig. 4. MW, molecular weight.

Despite their recurrence, it remains unknown whether *KBTBD4* mutations drive malignancy in MB. To determine the essentiality of KBTBD4, we used base editing in patient-derived xenograft (PDX) models derived from group 3 MB tumour specimens harbouring either *KBTBD4-PR* or wild-type *KBTBD4* (*KBTBD4-WT*). We designed single-guide RNAs (sgRNAs) predicted to introduce missense hypomorphic mutations into KBTBD4 through an adenosine base editor (ABE8e); this included two sgRNAs that target both the WT and mutant *KBTBD4* alleles (sgY120 and sgC297) as well as one sgRNA that specifically edits the *KBTBD4-PR* allele proximal to the insertion (sgM316) (Fig. 1a). The major base editing outcomes predicted for sgY120 and sgC297 are Y120H and C297R, respectively, whereas for sgM316 it is M316T/W317R in KBTBD4-PR. Owing to technical limitations and sensitivity of the PDX cells to other delivery methods, we packaged ABE8e ribonucleoproteins into engineered virus-like particles (eVLPs)^22^. We first tested the efficiency of eVLPs containing these sgRNAs as well as the control sgRNA, sgHEK3, in K562 cells, verifying that sgY120 and sgC297 eVLPs led to significant KBTBD4 depletion, probably owing to protein destabilization (Extended Data Fig. 1a). We then verified high editing efficiency of eVLPs containing sgY120, sgC297 and sgHEK3 after transduction in ICB1299, a PDX model that harbours *KBTBD4-PR* that can be effectively transduced and uniquely expanded ex vivo, as well as CHLA-01-MED (*KBTBD4-WT*) and MED411FH-TC (*KBTBD4-WT*), albeit to a lesser extent (Extended Data Fig. 1b). As expected, sgM316 led to editing of the *KBTBD4-PR* allele only in ICB1299. Notably, the KBTBD4-targeting eVLPs, including sgM316, inhibited the ex vivo proliferation of ICB1299, whereas the effects were minimal in the WT models (Fig. 1b). *KBTBD4* is not designated as a common essential gene from the Cancer Dependency Map (DepMap) (Extended Data Fig. 1c)^23^. Altogether, we establish that the *KBTBD4-PR* mutation is required for the proliferation of a KBTBD4-mutant group 3 MB PDX model.

## MB mutants potentiate CoREST degradation

To determine how *KBTBD4* mutations affect the MB proteome, we conducted global proteomics in group 3 PDX models, including two harbouring the *KBTBD4*-*PR* mutation and five with *KBTBD4-WT*. Comparison of the WT and PR mutant models identified 64 and 82 proteins that were significantly up- and downregulated, respectively, in the mutant samples (Fig. 1c and Supplementary Data 1 and 2). Functional network analysis of the differentially expressed proteins revealed that a network of HDAC2-associated proteins was depleted (Fig. 1d and Extended Data Fig. 1d,e). Most notably, this included members of the CoREST corepressor complex (that is, RCOR1, hereafter referred to as CoREST, RCOR2, RCOR3, LSD1)^5^. Importantly, HDAC1 is a paralogue of HDAC2 that can interchangeably associate as a member of the LHC complex. Co-immunoprecipitation experiments with HA–KBTBD4-PR efficiently retrieved LSD1, CoREST, HDAC1 and HDAC2, showing that either paralogue, HDAC1 or HDAC2, can associate with mutant KBTBD4 (Fig. 1e). Inactivation of KBTBD4-PR by base editing in ICB1299 increased levels of CoREST and LSD1 (Fig. 1f), indicating that KBTBD4-PR mediates their degradation. By contrast, levels of HDAC1 and HDAC2 did not substantially increase with KBTBD4-PR base editing, consistent with their modest reduction in KBTBD4-mutant PDX models by global proteomics (Fig. 1c). Altogether, these results demonstrate that the HDAC1/2-associated corepressors, LSD1 and CoREST, are selectively depleted in clinically relevant KBTBD4-mutant PDX models of group 3 MB.

**Extended Data Fig. 2:**
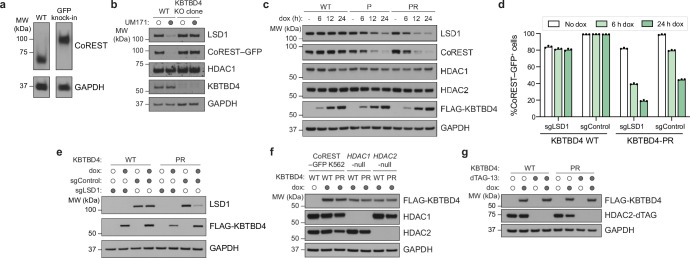
Supporting data for inducible KBTBD4 overexpression experiments. **a**, Immunoblots showing CoREST and GAPDH for WT and CoREST–GFP K562 cells. **b**, Immunoblots showing LSD1, CoREST–GFP, HDAC1, KBTBD4, and GAPDH in WT K562 CoREST–GFP knock-in cells and a clonal cell line with KBTBD4 knockout treated with DMSO or UM171 (1 µM) for 6 h. **c**, Immunoblots of indicated proteins in K562 *KBTBD4*-null CoREST–GFP cells after dox-inducible overexpression of indicated FLAG-KBTBD4 variants. **d**, Flow cytometry quantification of GFP^+^ cells for K562 CoREST–GFP cells. Data are mean ± s.d. of *n* = 3 biological replicates. **e**, Immunoblots showing LSD1, FLAG-KBTBD4, and GAPDH for K562 CoREST–GFP cells transduced with Cas9 and indicated sgRNAs and with dox-inducible overexpression of indicated KBTBD4 variants. **f**, Immunoblots showing HDAC1, HDAC2, FLAG-KBTBD4, and GAPDH for indicated K562 CoREST–GFP cells after dox-inducible overexpression of indicated FLAG-KBTBD4 variants (24 h). **g**, Immunoblots showing HDAC2-dTAG, FLAG-KBTBD4, and GAPDH for K562 CoREST–GFP *HDAC1*-null HDAC2-dTAG cells after dox-inducible overexpression of indicated KBTBD4 variants (24 h). *HDAC1*-null HDAC2-dTAG cells were pre-treated with DMSO or dTAG-13 (500 nM, 2 h). Data in **a**-**g** are representative of two independent experiments. FACS-gating schemes and uncropped blots can be found in Supplementary Figs. 1a, 5, respectively. MW, molecular weight. Source data

We established K562 cell lines with doxycycline (dox)-inducible expression of either KBTBD4-P or KBTBD4-PR. We initially focused on these two mutants as they are the most frequent clinical variants. Additionally, these cell lines contain GFP knocked in-frame at the CoREST C terminus and knockout of endogenous KBTBD4 (referred to as CoREST–GFP/*KBTBD4*-null cells) (Extended Data Fig. 2a,b). A time course of mutant KBTBD4 dox-induced expression demonstrated that both mutant ligases cause degradation of CoREST and LSD1 (ref. ^5^), with more rapid and potent degradation observed with KBTBD4-PR (Fig. 1g and Extended Data Fig. 2c). By comparison, substantial depletion of HDAC1/2 was not observed in this time frame. CoREST degradation was blocked by addition of MLN4924, a neddylation inhibitor that disrupts the activity of CULLIN-RING E3 ligases (Fig. 1g). Next, we sought to determine whether LSD1, HDAC1 and/or HDAC2 are necessary for CoREST degradation. Although knockout of LSD1 had minimal effect on CoREST degradation, individual knockout of HDAC1 or HDAC2, separately, caused partial rescue (Fig. 1h and Extended Data Fig. 2d–f). The partial effects of the HDAC1 and HDAC2 single knockouts suggest that the paralogues are functionally redundant in mediating CoREST degradation. To test this notion, we generated an *HDAC1*-null cell line containing endogenous HDAC2-dTAG knock-in, which permits conditional HDAC1/2 double knockout upon dTAG-13 treatment^24,25^. In the *HDAC1*-null context, HDAC2 depletion further rescued CoREST degradation by KBTBD4-PR dox-induced expression (Fig. 1i and Extended Data Fig. 2g). Altogether, our results demonstrate that HDAC1 and HDAC2 are critical for CoREST degradation by KBTBD4-PR and are functionally redundant in this mechanism.

**Extended Data Fig. 3:**
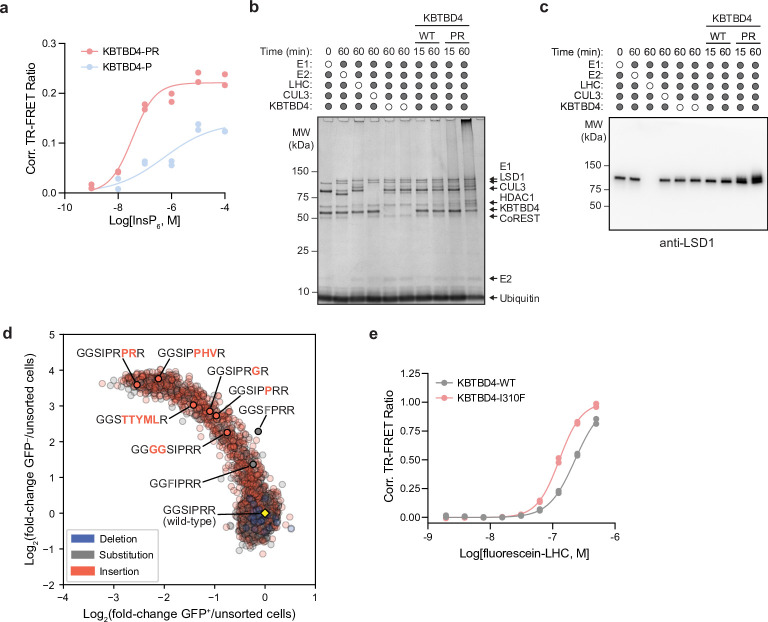
Supporting data for in vitro biochemical experiments and DMS. **a**, TR-FRET signal between fluorescein-LHC and anti-His CoraFluor-1-labelled antibody with indicated His-KBTBD4 variant in the presence of varying concentrations of InsP_6_ (*n* = 2 biological replicates). **b**, Coomassie staining for in vitro ubiquitination assays of either CRL3^KBTBD4-WT^ and CRL3^KBTBD4-PR^ with LHC. **c**, Immunoblot for LSD1 of in vitro ubiquitination assays of either CRL3^KBTBD4^ and CRL3^KBTBD4-PR^ with LHC. **d**, Scatterplot showing log_2_(fold-change) enrichment of KBTBD4 variants in GFP^+^ and in GFP^–^ cells versus unsorted cells normalized to WT. Deletion, substitution, and insertion variants are colored in blue, gray, and red, respectively, and WT is marked by a yellow diamond. MB mutant sequences are labeled. Pearson correlation coefficient *r* = −0.91, two-sided *P* value < 10^−307^. Data are mean of *n* = 3 biological replicates and the overall DMS experiment was performed once. **e**, TR-FRET signal between fluorescein-LHC and anti-His CoraFluor-1-labelled antibody with indicated His-KBTBD4 variant in the presence of InsP_6_ (50 µM) (*n* = 2 biological replicates). Data in **a** and **e** are representative of two independent experiments. Data in **b** and **c** are representative of *n* = 3 biological replicates. FACS-gating schemes and uncropped blots can be found in Supplementary Figs. 1a, 6, respectively. Corr., corrected; MW, molecular weight. Source data

We next sought to establish whether the KBTBD4-mutant E3 ligase directly engages and ubiquitinates the LHC complex using a reconstituted biochemical system^26,27^ (Methods). We first measured their association in vitro using time-resolved Förster resonance energy transfer (TR-FRET)^28,29^ (Fig. 1j). Notably, KBTBD4-P and KBTBD4-PR both demonstrated greater affinity with LHC than KBTBD4-WT, showing that the MB mutants are sufficient to drive E3 engagement with LHC. KBTBD4-PR exhibited stronger binding to LHC than KBTBD4-P, consistent with our co-immunoprecipitation and CoREST degradation experiments (Fig. 1e,g). Critically, mutant KBTBD4–LHC binding required the addition of inositol hexakisphosphate (InsP_6_) (Extended Data Fig. 3a), a cofactor that stabilizes the PPI between HDAC1/2 and its cognate corepressors^30^, including CoREST. Lastly, reconstituted CRL3^KBTBD4-PR^ exhibited increased ubiquitination of CoREST in vitro in comparison with CRL3^KBTBD4-WT^ (Fig. 1k and Extended Data Fig. 3b,c). Together, these data demonstrate that *KBTBD4* MB mutations are sufficient to increase ubiquitination and degradation of CoREST in an HDAC1/2-dependent fashion.

## 2b-2c loop deep mutational scanning

Point substitutions and indels can incur fundamentally different impacts on protein structure and function, with the latter typically having more severe effects^31–33^. In the case of KBTBD4, both point substitutions and diverse indels can occur in group 3 and 4 MB, with a marked bias towards the latter^3^. The reason for this bias is not fully understood, possibly reflecting the increased likelihood of indels to promote gain-of-function interactions or resulting from the error-prone processes (that is, copy count variants from faulty DNA replication) that could favour their formation^34,35^.

**Fig. 2:**
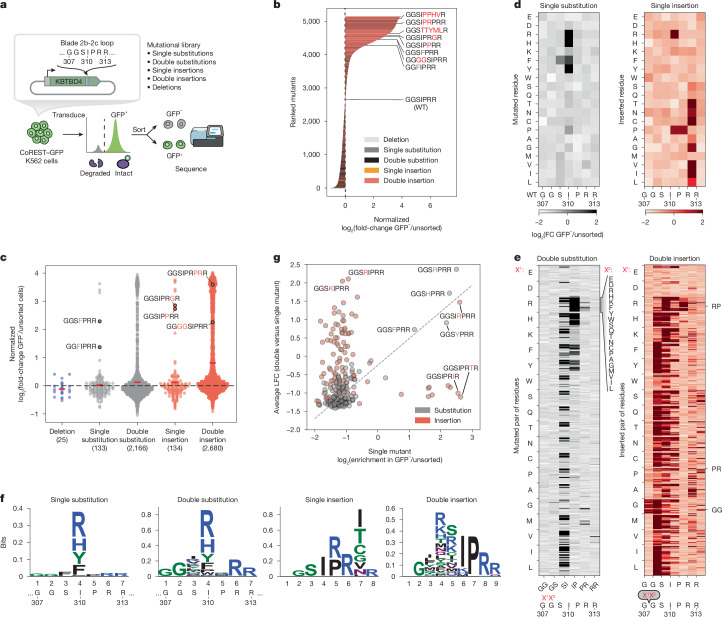
DMS of the 2b-2c loop. **a**, Schematic of the KBTBD4 2b-2c loop DMS. **b**, Waterfall plot displaying KBTBD4 variants ranked by log_2_(fold-change) enrichment in GFP^−^ over unsorted population normalized to WT. **c**, Swarm plot showing log_2_(fold-change) enrichment of KBTBD4 variants in GFP^−^ cells versus unsorted cells, normalized to WT and classified by mutation type. Dotted line indicates log_2_(fold-change) enrichment of WT KBTBD4 and solid black bars indicate mean for each mutation type. **d**, DMS for single substitutions (left) and single insertions (right) displayed as heatmaps of log_2_(fold-change) enrichment in GFP^−^ cells. **e**, DMS for double substitutions (left) and double insertions (right) displayed as heatmaps of log_2_(fold-change) enrichment in GFP^−^ cells. The *x* axis indicates the positions of the mutated amino acid pairs (X^1^X^2^), with identities of the substituted or inserted residues shown on the *y* axis. Specifically, the first substituted or inserted residue (X^1^) is indicated by the lefthand labels, whereas the second residue (X^2^) is indicated by each row. **f**, Sequence logo depicting relative entropy of amino acids at each position for single substitution, double substitution, single insertion and double insertion mutant sequences. Amino acids are coloured by their chemical characteristics: hydrophobic (black), polar (green), basic (blue), acidic (red) and neutral (purple). **g**, Scatterplot showing log_2_(fold-change) enrichment of single mutant KBTBD4 variants at the *n*th position (either substitution or insertion) in GFP^−^ cells (*x* axis) and average fold-change of the corresponding double mutants created by mutation of the adjacent *n* − 1 or *n* + 1 position (*y* axis). Linear correlations (dotted line) on the basis of linear least-squares regression for the substitutions are displayed on the plot (Pearson correlation coefficient *r* = 0.856, two-sided *P* = 1.74 × 10^−39^). Data in **b**–**g** are mean of *n* = 3 biological replicates and the overall DMS experiment was performed once. FACS-gating schemes are shown in Supplementary Fig. 1b. Schematic in **a** adapted from ref. ^43^, Springer Nature America.

To address these possibilities, we used deep mutational scanning (DMS) to profile the mutational landscape of the 2b-2c loop and systematically compare the effects of point substitutions and indels on CoREST neodegradation. Specifically, we constructed a library of KBTBD4 mutants (5,240 total) comprising all possible amino acid deletions (25), single amino acid substitutions (133), double amino acid substitutions involving pairs of adjacent residues (2,166), single amino acid insertions (134) and double amino acid insertions (2,680) across the 7-amino acid sequence spanning Gly307 and Arg313; as well as 100 randomly scrambled WT sequences and the two remaining MB indels (PR311delinsPPHV, IPR310delinsTTYML) not encompassed in the aforementioned categories (Fig. 2a and Supplementary Data 3 and 4). This mutant pool was transduced into CoREST–GFP/*KBTBD4*-null cells and, after 3 days, GFP^−^ and GFP^+^ cells were sorted by fluorescence-activated cell sorting (FACS). The identities of enriched and depleted KBTBD4 variants were then determined using next-generation sequencing and compared with variant frequencies from transduced, unsorted cells (Fig. 2b).

As anticipated, KBTBD4 variants enriched in GFP^−^ cells (that is, CoREST–GFP degraded) were depleted in GFP^+^ cells (that is, CoREST–GFP intact) (Extended Data Fig. 3d). Reassuringly, the KBTBD4-WT sequence was depleted in GFP^−^ cells, whereas the MB mutations were enriched to varying degrees, with the insertion MB mutants generally outperforming the MB point substitutions in promoting neodegradation. Although deletions had minimal effect on CoREST–GFP degradation, both substitutions and insertions could significantly promote neodegradation—with the double amino acid perturbations generally outperforming the single amino acid perturbations (Fig. 2c). In particular, the double insertions as a class were the most effective at promoting CoREST–GFP neodegradation overall (Fig. 2b,c), in agreement with their biased enrichment as MB mutations.

**Extended Data Fig. 4:**
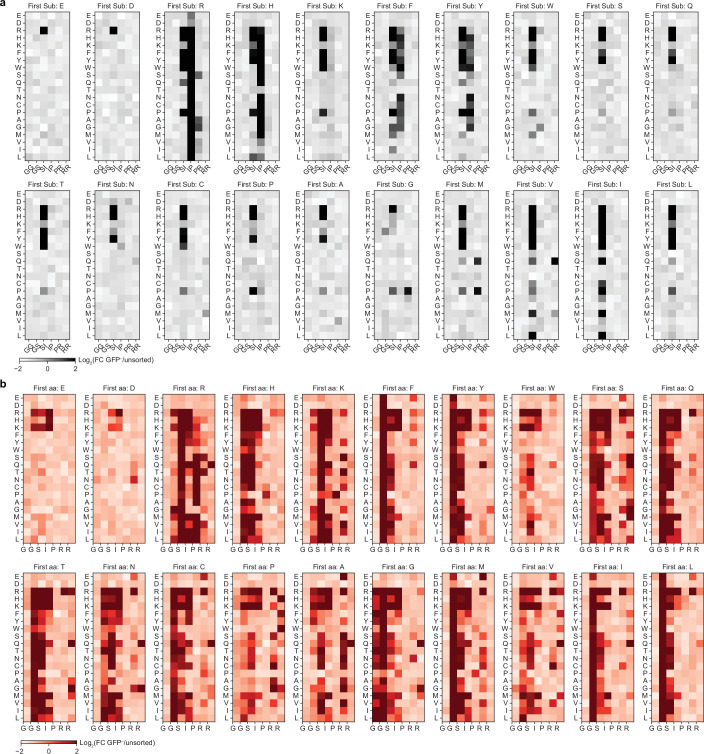
Supporting data for double-substitution and -insertion mutational scanning. **a**, Double-substitution deep mutational scanning displayed as heatmaps of log_2_(fold-change) enrichment in GFP^–^ cells versus unsorted cells for each possible pair of mutated amino acids. **b**, Double-insertion deep mutational scanning displayed as heatmaps of log_2_(fold-change) enrichment in GFP^–^ cells versus unsorted cells for each possible pair of mutated amino acids. Color intensity represents mean of *n* = 3 biological replicates and the overall DMS experiment was performed once.

We next scrutinized the amino acid backbone positions and side chain alterations in each mutant category that most effectively enhanced CoREST–GFP neodegradation. Efficacious single substitutions highly favoured mutations at position 4 (that is, Ile310) to bulkier positively charged (Arg and His) or aromatic amino acids (Phe and Tyr) (Fig. 2c,d), including the I310F MB mutant—which we also verified shows enhanced complexation with LHC by TR-FRET (Extended Data Fig. 3e). This positional and amino acid preference was maintained for the double substitutions, for which mutation of Ile310 (that is, second position of Ser309-Ile310 or first position of Ile310-Pro311) was highly favoured (Fig. 2e,f and Extended Data Fig. 4a). In fact, the most effective double substitutions were derived from the most effective single substitutions by further mutation of an adjacent position (that is, Ser309 or Pro311) (Fig. 2g, Pearson’s *r* = 0.856, *P* value = 1.74 × 10^−39^). By contrast, efficacious single insertions showed less positional and amino acid bias (Fig. 2d,f), albeit insertions after position 6 (that is, Arg312) were favoured. Amino acids inserted after position 6 could be diverse, suggesting that the primary function of the insertion may be to shift the 2b-2c loop so that Arg312 is moved to position 5. Supporting this notion, insertion of an Arg residue after position 4 (that is, Ile310) was uniquely potent for CoREST–GFP neodegradation, which, together with our single substitution data, suggests that introduction of an Arg residue in the middle of the loop is heavily favoured.

Notably, effective double insertions significantly diverged from the single insertions, showing a stronger preference for insertion earlier in the 2b-2c loop sequence (Fig. 2e,f and Extended Data Fig. 4b). This positional preference was less pronounced when Arg, and to a lesser extent Lys, Met and Pro, constituted one of the inserted amino acids. Notably, effective double insertions could tolerate many types of amino acids, except acidic residues (Asp and Glu) or Trp. These relaxed preferences probably explain the enhanced performance of double insertions in the deep mutational scan (Fig. 2c), suggesting they may operate to introduce a bulkier or basic amino acid into the loop either by shifting the position of Ile310 or through direct insertion of such a residue. In contrast to the relationship between single and double substitutions, the best double insertions were generally not derived from the most effective single insertions (that is, by insertion of an adjacent residue) (Fig. 2g, Pearson’s *r* = −0.04, not significant). These data suggest that (1) single and double insertions remodel the 2b-2c loop by distinct mechanisms, in contrast to single and double substitutions, and that (2) insertions are particularly effective in promoting gain-of-function PPIs^32^. Altogether, our DMS supports the notion that double insertions are enriched in KBTDB4-mutant MBs because of their propensity to promote neodegradation.

## Overall structures of mutant KBTBD4–LHC

**Fig. 3:**
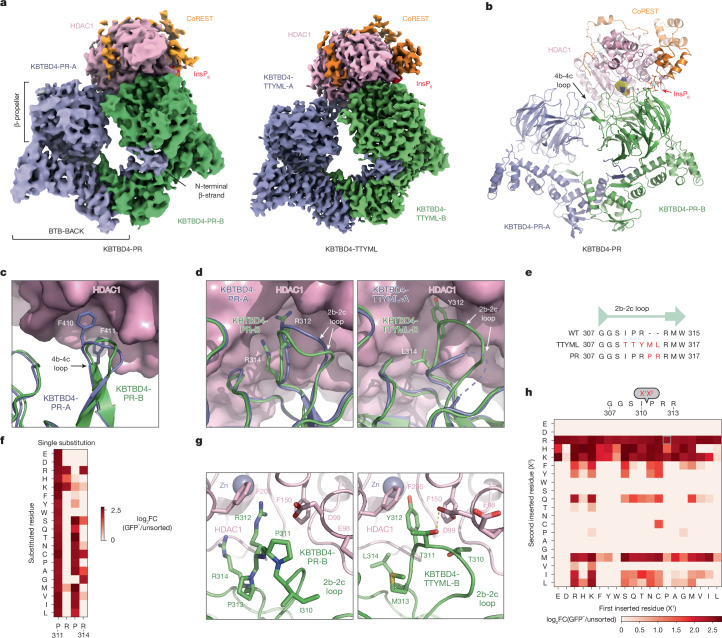
Structural mechanisms and amino acid preferences of functional KBTBD4 mutations. **a**, Cryo-EM map of LHC-bound KBTBD4 mutants with the two KBTBD4 protomers (slate and green), HDAC1 (pink), CoREST (orange) and InsP_6_ (red). Left, KBTBD4-PR; right, KBTBD4-TTYML. **b**, Ribbon diagram of the KBTBD4-PR–HDAC1–CoREST–InsP_6_ complex. Subunits of the complex are coloured the same way as in **a**. The hotspot arginine residue is shown in space filling model mode. InsP_6_ is shown in cyan and red sticks. **c**, Close-up view of the 4b-4c loops of KBTBD4-PR-A (slate) and KBTBD4-PR-B (green) after the β-propeller domain of the latter is superimposed onto that of the former. Side chains of two phenylalanine residues in the 4b-4c loop of KBTBD4-PR-A are shown in sticks. **d**, Close-up view of the 2b-2c loops of KBTBD4-A (slate) and KBTBD4-B (green) after the β-propeller domain of the former is superimposed onto that of the latter. Side chains of two arginine residues in the 2b-2c loop of KBTBD4-B are shown in sticks. Left, KBTBD4-PR; right, KBTBD4-TTYML. **e**, Alignment of KBTBD4-WT, PR and TTYML 2b-2c loop sequences. **f**, Single substitution DMS displayed as a heatmap of log_2_(fold-change) enrichment in GFP^−^ cells for each mutated amino acid in the KBTBD4-PR PRPR sequence. **g**, Close-up view of the interface between HDAC1 (pink) and the 2b-2c loop of KBTBD4-B (green) with the side chains of key residues shown in sticks. Left, KBTBD4-PR; right, KBTBD4-TTYML. **h**, Double insertion DMS displayed as a heatmap of log_2_(fold-change) enrichment in GFP^−^ cells for each pair of mutated amino acids (X^1^, X^2^) inserted after Ile310. Data in **f** and **h** are mean of *n* = 3 biological replicates and the overall DMS experiment was performed once.

**Extended Data Fig. 5:**
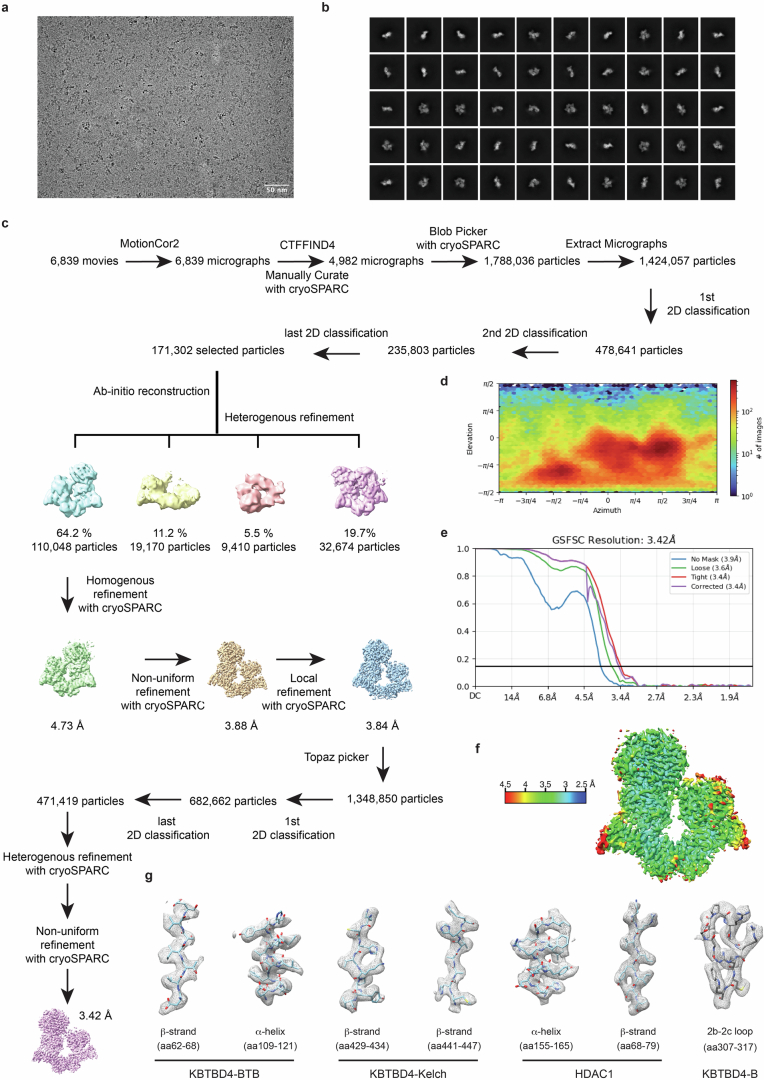
Cryo-EM data processing for the KBTBD4-PR-LHC complex. **a**, A representative cryo-EM micrograph out of 4,982 micrograph; scale bar, 50 nm. **b**, Typical 2D averages of the cryo-EM dataset. **c**, Flowchart of single particle analysis of the KBTBD4-PR-LHC complex. **d**, Angular distribution of particles used in the final reconstruction. **e**, Fourier shell correlation (FSC) curves for KBTBD4-PR-LHC. At the Gold-standard threshold of 0.143, the resolution is 3.42 Å. **f**, Local resolution map of the KBTBD4-PR-LHC complex from 2.5 to 4.5 Å. **g**, Density maps of representative regions of the KBTBD4-PR-LHC complex fit with the structural model shown in sticks.

**Extended Data Fig. 6:**
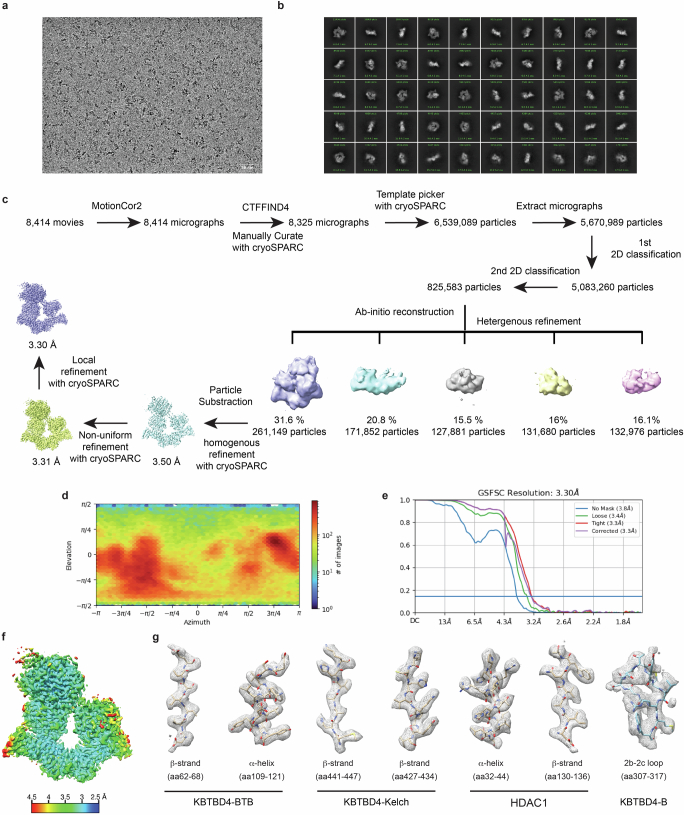
Cryo-EM data processing for the KBTBD4-TTYML-LHC complex. **a**, A representative cryo-EM micrograph out of 8,325 micrographs; scale bar, 50 nm. **b**, Typical 2D averages of the cryo-EM dataset;. **c**, Flowchart of single particle analysis of the KBTBD4-TTYML-LHC complex. **d**, Angular distribution of particles used in the final reconstruction. **e**, Fourier shell correlation (FSC) curves for KBTBD4-TTYML-LHC. At the Gold-standard threshold of 0.143, the resolution is 3.30 Å. **f**, Local resolution map of the KBTBD4-TTYML-LHC complex from 2.5 to 4.5 Å. **g**, Density maps of representative regions of the KBTBD4-TTYML-LHC complex fit with the structural model shown in sticks.

To elucidate the mechanism by which double insertions enable high-affinity engagement of the LHC complex, we determined the cryo-electron microscopy (cryo-EM) structures of two LHC-bound KBTBD4 MB mutants, KBTBD4-PR and KBTBD4-TTYML. We chose KBTBD4-TTYML in addition to KBTBD4-PR mutant because it lacks a basic residue in the 2b-2c loop. The KBTBD4 dimer is well resolved in both structures, which were determined at 3.42 and 3.30 Å resolution for the PR and TTYML mutants, respectively (Fig. 3a,b, Extended Data Figs. 5 and 6 and Extended Data Table 1). The three-dimensional (3D) reconstruction maps enabled us to trace the entire E3 polypeptide in both structures with high confidence.

**Extended Data Fig. 7:**
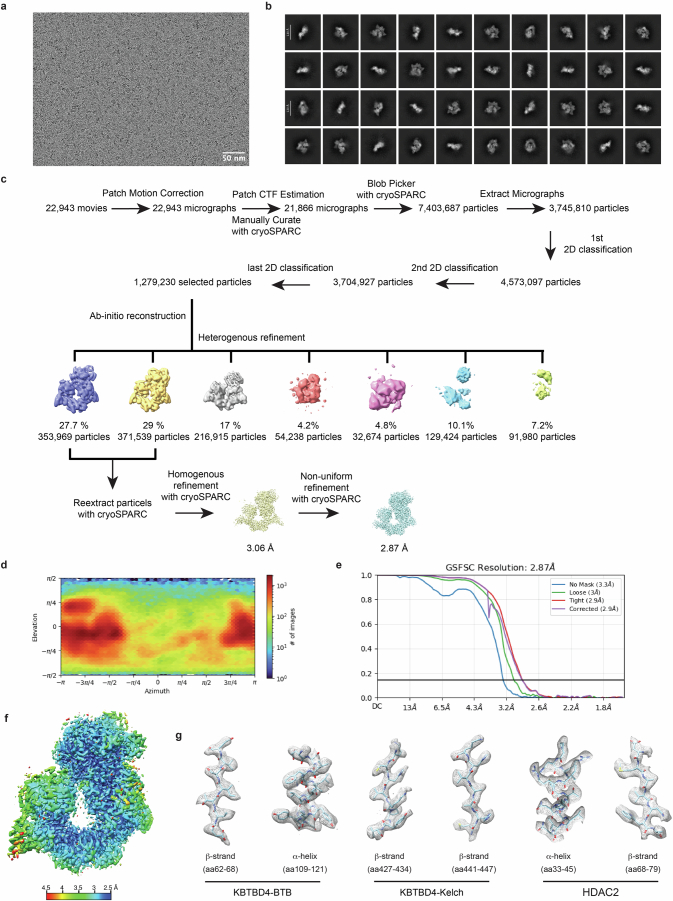
Cryo-EM data processing for the KBTBD4-PR-HDAC2-CoREST complex. **a**, A representative cryo-EM micrograph out of 21,866 micrographs; scale bar, 50 nm. **b**, Typical 2D averages of the cryo-EM dataset. **c**, Flowchart of single particle analysis of the KBTBD4-PR-HDAC2-CoREST complex. **d**, Angular distribution of particles used in the final reconstruction. **e**, Fourier shell correlation (FSC) curves for KBTBD4-PR-HDAC2-CoREST. At the Gold-standard threshold of 0.143, the resolution is 2.87 Å. **f**, Local resolution map of the KBTBD4-PR-HDAC2-CoREST complex from 2.5 to 4.5 Å. **g**, Density maps of representative regions of the KBTBD4-PR-HDAC2-CoREST complex fit with the structural model shown in sticks.

**Extended Data Fig. 8:**
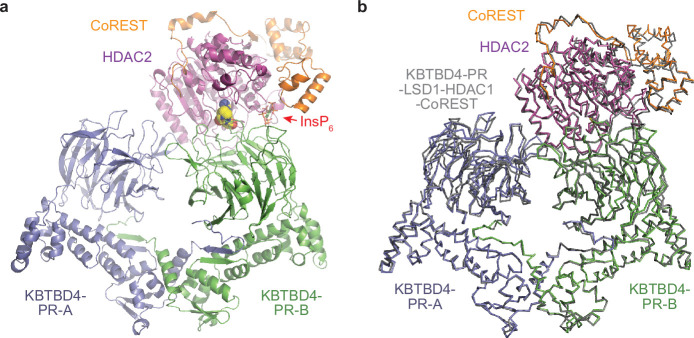
Cryo-EM structure of the KBTBD4-PR-HDAC2-CoREST complex. **a**, Overall structure of the KBTBD4-PR-HDAC2-CoREST complex. **b**, Superposition of KBTBD4-PR-LHC and KBTBD4-PR-HDAC2-CoREST complexes.

As expected, KBTBD4 adopts a well-defined dimeric architecture with pseudo-two-fold symmetry (Fig. 3a,b). In a head-to-head fashion, both KBTBD4 mutants homodimerize through the N-terminal BTB domain with the C-terminal KELCH-repeat domain positioned atop the elongated BTB-BACK structure module. Despite their apparent two-fold symmetry, the two mutant KBTBD4 dimers bind LHC in an asymmetric, concerted assembly. In both cryo-EM maps, only HDAC1 and part of the ELM2 and SANT1 domains of CoREST are well resolved. The rest of the LHC complex is presumably too flexible to be visualized by 3D reconstruction. Indeed, we were able to assemble a complex formed among KBTBD4-PR, HDAC2 and CoREST, and determined its cryo-EM structure at 2.87 Å resolution (Extended Data Fig. 7 and Extended Data Table 1). Superposition analysis indicates that CoREST-bound HDAC2 is docked to the KBTBD4-PR mutant in a manner nearly identical to HDAC1 (Extended Data Fig. 8), confirming the functional redundancy of HDAC1 and HDAC2 in the mutant KBTBD4 mechanism. Our subsequent structural analyses focus on the two HDAC1-containing complexes.

HDAC1 is the only subunit of LHC that makes direct contact with the KBTBD4 mutants. In both E3–neosubstrate assemblies, the KBTBD4 dimer engages LHC by asymmetrically cupping the catalytic domain of HDAC1 through its two β-propellers. Unexpectedly, in contrast to other KELCH CRL3s (ref. ^36^), the mutant E3 dimers do not use the top surface of their β-propellers to recognize HDAC1. In the KBTBD4-PR mutant dimer, one E3 protomer, designated as KBTBD4-PR-A, uses the 4b-4c loop in its β-propeller to latch onto the edge of the HDAC1 catalytic domain, whereas the other protomer, KBTBD4-PR-B, more extensively wraps around the HDAC1 active site through a lateral surface region of its KELCH-repeat domain (Fig. 3b). Notably, next to the HDAC1 active site, a molecule of InsP_6_ makes direct contacts with HDAC1, CoREST and KBTBD4, acting as a molecular glue to stabilize the protein–protein interfaces (Extended Data Fig. 3a). The two β-propellers of KBTBD4, therefore, use distinct structural elements to asymmetrically engage the deacetylase.

## Mechanisms of E3 neomorphic mutations

**Extended Data Fig. 9:**
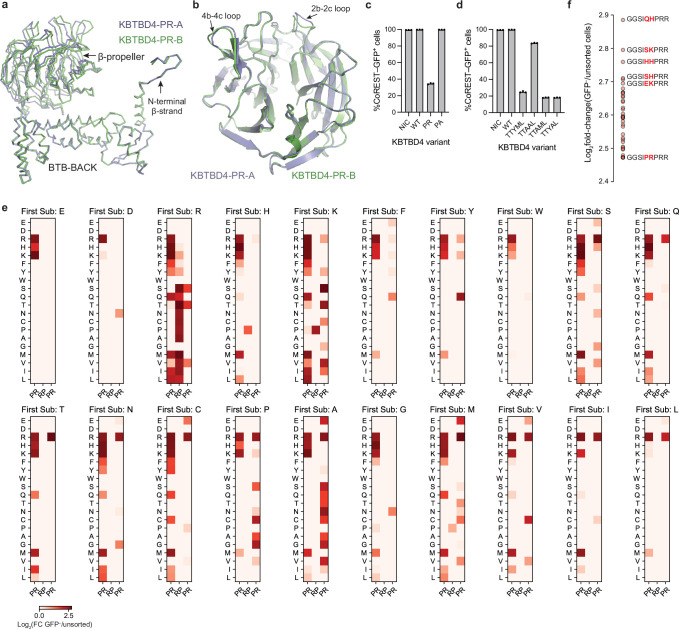
Structural and functional analysis of mutant KBTBD4-LHC complexes. **a**, Superposition of the two KBTBD4-PR protomers in complex with HDAC1. The two protomers, KBTBD4-PR-A (slate) and KBTBD4-PR-B (green) are superimposed via their BTB-BACK domain. **b**, Superposition of the KELCH-repeat domains of the two KBTBD4-PR protomers (KBTBD4-PR-A: slate; KBTBD4-PR-B: green) in complex with HDAC1. Noticeable structural differences at the 2b-2c and 4b-4c loops are indicated. **c**,**d**, Flow cytometry quantification of GFP^+^ cells for *KBTBD4*-null CoREST–GFP cells after overexpression of indicated KBTBD4 variant. **e**, Double-substitution deep mutational scanning displayed as heatmaps of log_2_(fold-change) enrichment in GFP^–^ cells versus unsorted cells for each possible pair of mutated amino acids in the KBTBD4-PR PRPR sequence. Color intensity represents mean of *n* = 3 biological replicates. **f**, Top-enriched double-insertion mutants inserted after Ile310, more effective than KBTBD4-PR, ranked by their log_2_(fold-change) enrichment in GFP^–^ over unsorted population. Data in **c** and **d** are mean ± s.d. of *n* = 3 technical replicates and representative of two independent experiments. Data in **e** and **f** are mean of *n* = 3 biological replicates and the overall DMS experiment was performed once. FACS-gating schemes can be found in Supplementary Fig. 1a. NIC: non-infection control. Source data

Superposition analysis of the two KBTBD4-PR protomers indicates that the relative position of the β-propeller domain and the BTB-BACK domain is not identical in the two E3 chains (Extended Data Fig. 9a). The relative position of each β-propeller domain is likely optimized for binding HDAC1, consistent with a global induced-fit mechanism for assembling the E3–neosubstrate complex. Similar to the BTB-BACK module, the KELCH-repeat domain of each KBTBD4 mutant maintains the same structure throughout the entire β-propeller fold except at a few surface loop regions (Extended Data Fig. 9b). In particular, the 4b-4c loop and the 2b-2c loop, which are uniquely used by β-propeller-A and -B to interact with HDAC1, respectively, show clear structural deviations between the two protomers (Fig. 3c,d). In KBTBD4-TTYML, the mutant 2b-2c loop in β-propeller-A is solvent-exposed and structurally disordered. By contrast, the same loop in β-propeller-B adopts an ordered conformation, making close interactions with HDAC1 (Fig. 3d). Overall, the E3 dimer demonstrates both global and local structural plasticity to engage the neosubstrate.

The indel mutations in the PR and TTYML mutants elicit their gain-of-function effects at the centre of the HDAC1 β-propeller-B interface, representing the hotspot PPI site. The net effects of these two mutations are the expansion of the 2b-2c loop by two amino acids and, in the case of TTYML, alteration of the amino acid composition (Fig. 3e). The 2b-2c loops of the two KBTBD4 mutants are positioned at the periphery of the HDAC1 active site pocket and, despite their sequence diversity, both insert the bulky side chain of a central residue into the deacetylase catalytic site tunnel. For KBTBD4-PR, the positively charged side chain of Arg312 reaches halfway into the tunnel, whereas Arg314 occupies a nearby pocket at the tunnel entrance (Fig. 3d). In an analogous manner, the aromatic side chain of Tyr312 in KBTBD4-TTYML also protrudes into the HDAC1 tunnel, whereas Leu314 partially fulfils the same role as Arg314 in KBTBD4-PR (Fig. 3d). Mutation of Arg312 in KBTBD4-PR to alanine reduced degradation of CoREST (Extended Data Fig. 9c). By contrast, mutation of both Tyr312 and Met313 in KBTBD4-TTYML (that is, KBTBD4-TTAAL) was necessary to block CoREST degradation, suggesting that Met313 also has a critical role in the hotspot interaction or might functionally replace Tyr312 in the Tyr312Ala context (Extended Data Fig. 9d).

Taking advantage of our DMS data, we investigated the requirement of each residue in the central PRPR sequence of KBTBD4-PR for CoREST degradation (Fig. 3f,g). A basic residue is strongly preferred at the second position of this motif, corroborating the critical role of Arg312 at the interface. Arg314, however, can be replaced by smaller amino acids, suggesting an auxiliary function. The amino acid preferences at the two proline positions are more relaxed, consistent with their minor involvement in contacting HDAC1. However, if the first proline of the PRPR motif is replaced by a polar residue (X^1^ position), such as serine or lysine, the requirement for a basic amino acid at the X^2^ position is relaxed (Fig. 3h and Extended Data Fig. 9e). In this context, a hydrophobic or aromatic amino acid can now substitute the basic residue, presumably by inserting its bulky side chain into the HDAC1 active site tunnel. In fact, this notion is validated by the TTYML mutant, in which Tyr312 of the central TTYML motif has an equivalent role to Arg312 in the PRPR motif (Fig. 3g). Although tyrosine is not as strongly favoured as arginine (Fig. 3h), the two leading threonine residues, Thr310 and Thr311, of the TTYML mutant might compensate by making closer contacts with an HDAC1 surface loop—donating an H-bond from Thr311 to HDAC1 Asp99. These further interactions provide a plausible explanation for the relaxed requirement at the second position of the PRPR motif when the first proline is replaced by a polar residue and the superior activity of QHPR, SKPR and HHPR over PRPR in the deep mutational scan (Extended Data Fig. 9f). These synthetic mutants and the cancer mutants, therefore, exploit not only the active site tunnel of HDAC1, but also its peripheral regions. Taken together, the KBTBD4 gain-of-function mutants enable the interaction between the E3 and the neosubstrate by augmenting their shape complementarity and polar interactions.

## Molecular mimicry of mutant E3 and UM171

**Fig. 4:**
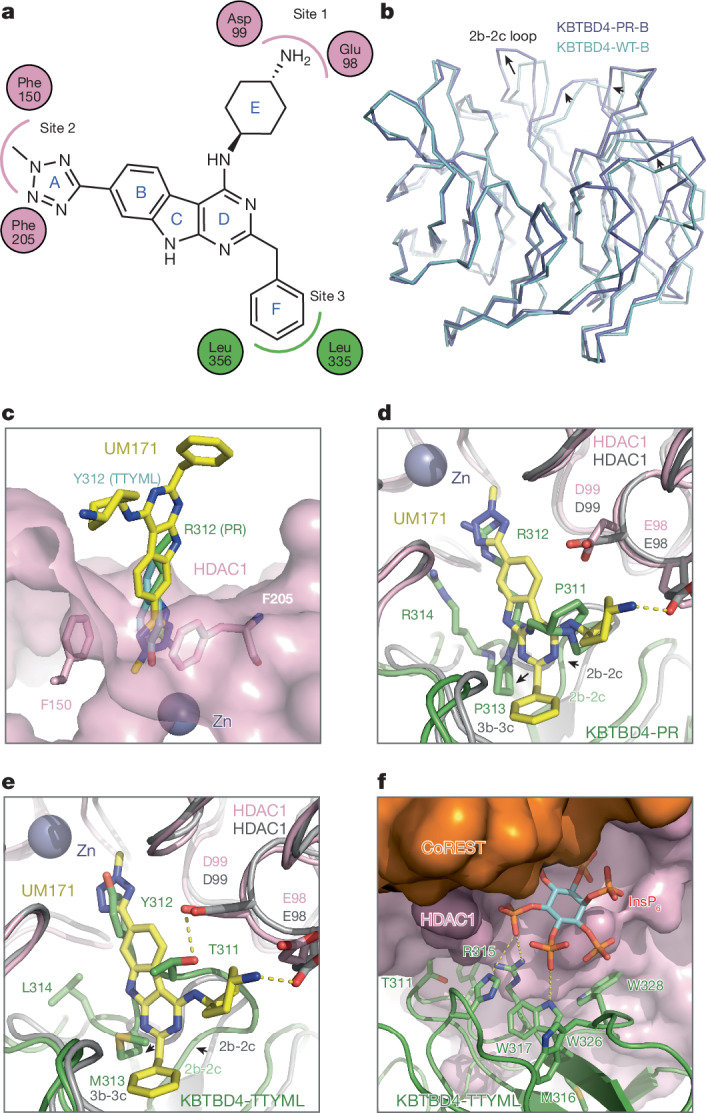
Converging mechanism between KBTBD4 cancer mutations and UM171. **a**, Simplified ligand plot of UM171–KBTBD4–HDAC1 interactions. HDAC1 and KBTBD4 residues are denoted by pink and green circles, respectively. **b**, Superposition analysis of the β-propellers in protomer-B of the KBTBD4-WT and KBTBD4-PR dimers. The structural differences at several top surface loops are indicated by arrows. The 2b-2c loop is labelled. **c**, A comparison of UM171 (yellow and blue sticks), the side chain of Tyr312 of KBTBD4-TTYML-B (cyan and red sticks) and the side chain of Arg312 of KBTBD4-PR-B (green and blue sticks) at the active site pocket of HDAC1 (pink). The three complex structures are superimposed through HDAC1. Two phenylalanine residues outlining the entrance of the HDAC1 active site tunnel are shown in sticks. **d**, A comparison between UM171 (yellow and blue sticks) and the 2b-2c loop of KBTBD4-PR-B with the KBTBD4–UM171–HDAC1 structure superimposed with the KBTBD4-PR–HDAC1 structure through HDAC1. The side chains of key residues at the interface are shown in sticks. **e**, A comparison between UM171 (yellow and blue sticks) and the 2b-2c loop of KBTBD4-TTYML-B with the KBTBD4–UM171–HDAC1 structure superimposed with the KBTBD4-TTYML–HDAC1 structure through HDAC1. The side chains of key residues at the interface are shown in sticks. **f**, A close-up view of the inter-molecular interfaces among KBTBD4-TTYML (green), HDAC1 (pink, surface representation), CoREST (orange, surface representation) and InsP_6_ (cyan, orange and red sticks). The side chains of key KBTBD4-TTYML residues involved in InsP_6_ interaction and at the nearby 2b-2c loop are shown in sticks. Zn, zinc.

Recent studies have shown that KBTBD4 is involved in the mechanism of action of UM171, a potent agonist of ex vivo haematopoietic stem cell expansion^21^ (Fig. 4a). Notably, UM171 phenocopies the *KBTBD4* MB mutations by promoting the ubiquitination and degradation of CoREST. In a companion study, we determined the structure of the KBTBD4–LHC complex stabilized by UM171 and demonstrated that the small molecule acts as a molecular glue to induce complex formation between the E3 and HDAC1 (ref. ^37^). A structural comparison between the KBTBD4–UM171–LHC complex and the two LHC-bound KBTBD4 MB mutants reveals a notable convergent mechanism by which the molecular glue and the cancer mutations complement and optimize the suboptimal protein–protein interface between the E3 ligase and HDAC1 to drive their association.

**Extended Data Fig. 10:**
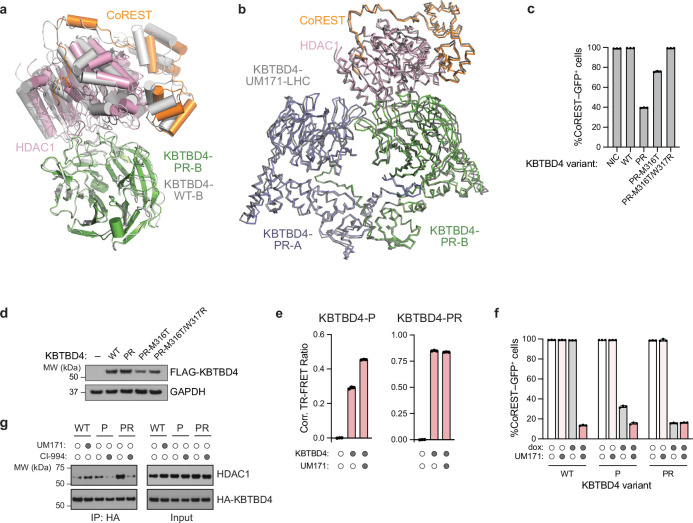
Structural and functional analysis of mutant KBTBD4-LHC and wild-type KBTBD4-LHC-UM171. **a**, A comparison of the relative positions between KBTBD4 β-propeller and the HDAC1-CoREST complex in the KBTBD4-UM171-LHC and KBTBD4-PR-LHC complex structures. The two complex structures are superimposed via the KELCH-repeat domain of KBTBD4-B. All subunits in the KBTBD4-UM171-LHC complex are colored in gray. UM171 and InsP_6_ are not shown. **b**, Superposition of the overall complex structures of KBTBD4-UM171-LHC and KBTBD4-PR-LHC. All subunits in KBTBD4-UM171-LHC are colored in gray. **c**, Flow cytometry quantification of GFP^+^ cells for *KBTBD4*-null CoREST–GFP cells after overexpression of indicated KBTBD4 variants generated by sgM316. Data are mean ± s.d. of *n* = 3 technical replicates. **d**, Immunoblots for FLAG-KBTBD4 and GAPDH in *KBTBD4*-null CoREST–GFP cells overexpressing indicated KBTBD4 variants. **e**, TR-FRET signal between fluorescein-LHC and anti-His CoraFluor-1-labelled antibody with indicated His-KBTBD4 variant in the presence of DMSO or UM171 (10 µM) (*n* = 2 biological replicates). **f**, Flow cytometry quantification of GFP^+^ cells for *KBTBD4*-null CoREST–GFP cells with dox-inducible overexpression of indicated KBTBD4 variant and treated with either DMSO or UM171 (1 µM) for 24 h. Data are mean ± s.d. of *n* = 3 biological replicates. **g**, Immunoblots of HA IP from 293T cells transfected with indicated HA-KBTBD4 variants, pre-treated with MLN4924 (1 µM) for 3 h, and then treated with DMSO or CI-994 (10 µM) for 3 h or UM171 (1 µM) for 1 h. Data in **c**-**g** are representative of two independent experiments. FACS-gating schemes and uncropped blots can be found in Supplementary Figs. 1a, 7, respectively. Corr., corrected; MW, molecular weight; NIC: non-infection control. Source data

The double insertion of the KBTBD4-PR mutant expands the 2b-2c loop and triggers subsequent conformational changes in several additional top surface loops across half of the β-propeller (Fig. 4b). Owing to these changes, the relative position of HDAC1–CoREST is slightly shifted when the KBTBD4–UM171–LHC complex is superimposed with the KBTBD4-PR–LHC complex through β-propeller-B (Extended Data Fig. 10a). Nonetheless, global superposition of the two complex structures can be made with a root mean squared deviation of 1.0 Å over approximately 1,500 Cα atoms, indicative of a highly similar overall architecture (Extended Data Fig. 10b). Indeed, the two E3–neosubstrate assemblies are stabilized by the same protein–protein interfaces and share common hotspot interactions centred around the HDAC1 active site pocket.

Closer inspection of the active site hotspot reveals that UM171 acts as a chemical mimetic of the *KBTBD4* MB mutations. Simplistically, UM171 possesses three ‘arms’, a cyclohexylamine (E ring), an *N-*methyltetrazole (A ring) and a benzyl group (F ring), which roughly occupy three sites that are also contacted by three distinct amino acids of mutant KBTBD4 (Fig. 4a). First, near the entrance of the pocket, the cyclohexylamine of UM171 and the X^1^ position of the two cancer mutants (that is, Pro311 of KBTBD4-PR and Thr311 of KBTBD4-TTYML) both contact the same HDAC1 loop comprising Asp99 and Glu98 (Fig. 4d,e, and site 1 Fig. 4a). Second, the tetrazole of UM171 mimics the side chain of the central amino acid at the mutants’ X^2^ position (for example, Arg312 of KBTBD4-PR and Tyr312 of KBTBD4-TTYML), where both protrude into the HDAC1 active site tunnel (Fig. 4c, and site 2 Fig. 4a). Lastly, whereas the benzyl group of UM171 induces and binds a surface groove between the 2b-2c and 3b-3c loops of KBTBD4 (site 3 Fig. 4a), the expanded 2b-2c loops of the two cancer mutants (that is, Pro313 of KBTBD4-PR and Met313 of KBTBD4-TTYML) occupy the same space (Fig. 4d,e). Importantly, the structural roles of UM171 and the double insertions in the two KBTBD4 mutants are mostly localized at the HDAC1 active site, allowing InsP_6_ to further strengthen the E3–neosubstrate interaction through a separate contact interface (Fig. 4f). Notably, eVLPs containing sgM316 edit Met316 and Trp317, the latter of which contacts InsP_6_, and mutation of this interface blocks CoREST degradation and probably drives the anti-proliferative effects of sgM316 in the *KBTBD4-PR* PDX model (Fig. 1b and Extended Data Fig. 10c,d). Lastly, UM171 partially synergized with KBTBD4-P but not KBTBD4-PR in enhancing engagement with LHC (Extended Data Fig. 10e,f), highlighting the plasticity of the induced protein interface. Altogether, the molecular glue and the gain-of-function cancer mutations structurally and functionally mimic each other, complementing the suboptimal protein–protein interface to promote the neomorphic interaction.

## HDAC1/2 inhibitors block mutant E3

**Fig. 5:**
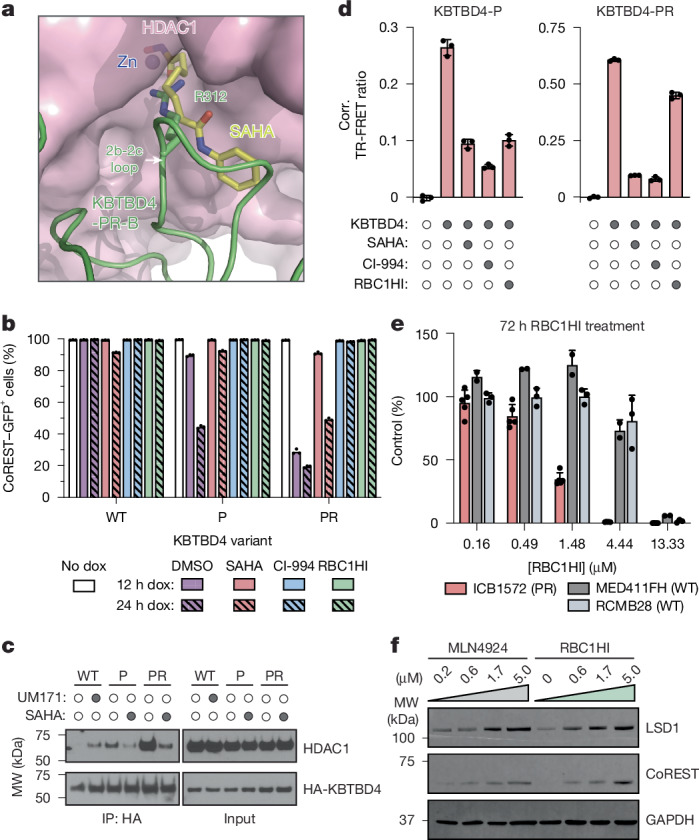
HDAC1/2 inhibitors block the neomorphic activity of KBTBD4 mutants. **a**, Steric clash between SAHA (yellow) and the central arginine residue at the 2b-2c loop of KBTBD4-PR-B. The KBTBD4-PR–HDAC1 complex structure is superimposed with the HDAC2–SAHA complex structure (PDB 4LXZ) through the HDAC subunits. **b**, Flow cytometry quantification of GFP^+^ cells for *KBTBD4*-null CoREST–GFP cells pre-treated with DMSO, CI-994 (10 µM), SAHA (10 µM) or RBC1HI (10 µM) for 1 h followed by dox-inducible overexpression of the indicated KBTBD4 variant. Data are mean ± s.d. of *n* = 3 biological replicates. **c**, Immunoblots of HA IP from 293T cells transfected with the indicated HA–KBTBD4 variant, pre-treated with MLN4924 (1 µM) for 3 h, and then treated with DMSO, UM171 (1 µM) or SAHA (10 µM) for 1 h. **d**, TR-FRET signal between fluorescein–LHC and anti-His CoraFluor-1-labelled antibody with indicated His–KBTBD4 mutant in the presence of DMSO, SAHA (10 µM), CI-994 (10 µM) or RBC1HI (10 µM) (*n* = 2 biological replicates). **e**, Ex vivo proliferation for ICB1572 (*KBTBD4-PR*), MED411FH (*KBTBD4-WT*) and RCMB28 (*KBTBD4-WT*) cells with RBC1HI treatment at indicated doses for 72 h. Data are mean ± s.d. across biological replicates from PDX cells derived from *n* = 5 (ICB1572), *n* = 3 (RCMB28) and *n* = 2 (MED411FH) implanted mice. **f**, Immunoblots showing LSD1, CoREST and GAPDH in ICB1572 after 24 h treatment with MLN4924 or RBC1HI at the indicated doses. Data in **b** and **d** and immunoblots in **c** and **f** are representative of two independent experiments. FACS-gating schemes and uncropped blots can be found in Supplementary Figs. 1a and 3, respectively. Source data

Our structural and DMS results reveal that KBTBD4 MB mutants promote CoREST degradation by directly engaging HDAC1/2 through a hotspot interaction in the deacetylase active site. These observations suggest that HDAC1/2 active site inhibitors may sterically occlude engagement by the mutant ligase, thereby blocking their oncogenic function. Superposition analysis of KBTBD4-PR–HDAC1 with HDAC2 bound to suberoylanilide hydroxamic acid (SAHA) supports the notion that an HDAC1/2 inhibitor and the inserted arginine residue might physically clash^38^ (Fig. 5a). In agreement, treatment with SAHA and CI-994, a 2-amino-benzamide-derived inhibitor^39^, could rescue CoREST degradation induced by KBTBD4-P and KBTBD4-PR expression (Fig. 5b). Furthermore, SAHA and CI-994 could block association of KBTBD4-P and KBTBD4-PR with HDAC1 in cells as well as in a purified reconstituted system (Fig. 5c,d and Extended Data Fig. 10g). We next tested RBC1HI, a recently developed selective HDAC1/2 inhibitor^40^. RBC1HI effectively blocked CoREST degradation but showed reduced inhibitory activity in our TR-FRET assay, especially against the more potent KBTBD4-PR mutant (Fig. 5b,d). Although seemingly incongruous, these findings are consistent with past studies showing that selective HDAC1/2 inhibitors possess slow-binding kinetics and often require disassociation of the corepressor complex to engage HDAC1/2 (refs. ^28,41^), which may not occur on the time scale of the in vitro experiments. Together, these findings support that HDAC1/2 inhibitors can disengage mutant KBTBD4 from HDAC1/2 to stabilize the associated corepressor complexes.

Our results suggest that HDAC1/2-selective inhibitors, such as RBC1HI, could selectively block the proliferation of KBTBD4-mutant MB tumour cells. To explore this concept, we evaluated the sensitivity of MB PDX models harbouring *KBTBD4-PR* to RBC1HI treatment. Ex vivo treatment of either *KBTBD4-WT* (MED411FH, RCMB28) or *KBTBD4-PR* mutant (ICB1572) PDX cells showed that the mutant displayed heightened sensitivity to RBC1HI (Fig. 5e). Moreover, ex vivo treatment of ICB1572 PDX cells with MLN4924 and RBC1HI, separately, led to increased levels of CoREST and LSD1 (Fig. 5f), consistent with KBTBD4 inhibition. Altogether, these results demonstrate the potential promise of selective HDAC1/2 inhibition as a strategy to block the proliferation of KBTBD4-mutant MB cells.

## Discussion

Here we elucidate the mechanism by which hotspot cancer mutations in the E3 ligase KBTBD4 can reprogramme its PPIs to promote aberrant degradation of HDAC1/2 corepressor complexes in MB, establishing HDAC1/2 as the target of mutant KBTBD4. Using DMS, we unveil the mutational landscape and molecular rules that control this neomorphic activity, highlighting how insertion mutations fundamentally differ from point substitutions in their preferences, effects and cooperativity, albeit in this specific E3 ligase context. Moreover, these findings underscore how insertions at a protein surface, in comparison with point substitutions, can be particularly effective at promoting neo-PPIs^32^. Leveraging these data with cryo-EM, we reveal the mechanistic basis by which MB mutations reconfigure the 2b-2c loop of KBTBD4 to engage the HDAC1 active site in a shape-complementary fashion. Notably, these further contacts made by the cancer mutations precisely mimic the effects of UM171 in gluing the suboptimal KBTBD4–HDAC1 interface, showcasing how chemical and genetic perturbations can act as molecular facsimiles. Understanding this molecular interface establishes the rationale for employing HDAC1/2 inhibitors to block the activity of KBTBD4 MB mutants and proliferation of *KBTBD4*-mutant PDX MB cells. Further study will be required to fully investigate the therapeutic potential of this approach and the downstream role of HDAC1/2 corepressor degradation in MB tumorigenesis.

We have previously shown that most molecular glues operate by potentiating weak, intrinsic interactions between two proteins^42^. In agreement, KBTBD4-WT shows low basal affinity towards LHC. By complementing the KBTBD4–HDAC1 interface, both UM171 and the MB cancer mutations can increase the protein binding affinity more than 5–25-fold. Similar to small molecule glues, cancer mutations, therefore, can also exploit and convert non-productive basal PPIs to confer neomorphic functions. This mechanistic mimicry between a molecular glue and human genetic mutations demonstrates how these perturbations can operate by similar molecular principles, and we anticipate that future instances of this chemical–genetic convergence may be uncovered and exploited for therapeutic applications. In conclusion, our study defines the mechanistic basis of E3 ligase gain-of-function cancer mutations and raises the prospect that massively parallel genetic methods may eventually enable de novo molecular glue discovery and design by identifying ‘glueable’ protein sites.

## Methods

### MB PDX lines

Ex vivo drug treatments, eVLP transduction and tandem-mass-tag (TMT)-proteomic study on PDXs were performed at St. Jude Children’s Research Hospital (SJCRH). NSG mice (NOD.Cg-*Prkdc*^*scid*^*Il2rg*^*tm1Wjl*^*/SzJ*; The Jackson Laboratory, JAX catalogue no. 005557) were used as hosts for PDX studies. Female NSG mice at least 8 weeks of age were anaesthetized in a surgical suite, and dissociated PDX cells were implanted in the cerebellum to amplify tumour material for downstream analyses. Mice were observed daily and euthanized at the onset of signs of sickness, including lethargy and neurological abnormalities. All clinical signs at the time of euthanasia did not exceed humane end point as determined by the SJCRH Institutional Animal Care and Use Committee (IACUC protocol no. 589-100536). RCMB51, RCMB52 and RCMB28 were originated and shared by R. J. Wechsler-Reya, Columbia University (previously Sanford Burnham Prebys). ICB1299 and ICB1572 were originated and shared by X.-N. Li, Northwestern University Feinberg School of Medicine (previously Baylor University). MED411FH, MED411FH-TC (established for tissue culture), MED211FH and MED2312FH were purchased from the Brain Tumor Research Laboratory, Seattle Children’s Hospital (previously Fred Hutchinson)^44^. Low passage PDXs (less than 10) were dissected and then flash-frozen for proteomics or dissociated for transduction and/or ex vivo drug sensitivity screening. Sample size choice was made to be at least *n* > 2 dissociated tumours for a given PDX model. No randomization of samples or blinding was conducted.

### Sample processing of mouse PDX tissues for TMT mass spectrometry

Frozen tissues (20–30 mg) from each mouse PDX tumour were added to 200 ml of freshly prepared 8 M urea lysis buffer (containing 12 g of urea, 10X HEPES in 25 ml of Millipore ultrapure water) and homogenized with glass beads in a Bullet Blender Tissue Homogenizer (Next Advance) for 5 min, followed by a 2-min centrifugation at 2,000 rpm. Subsequently, 1% sodium deoxycholate was immediately added to the lysed tissues and vortexed for 2 min, followed by centrifugation at 1,000 rpm. The resulting supernatants were collected and stored at −80 °C. For quality control and quantification, 2 ml of lysates from each sample were electrophoresed on 4–12% NuPAGE gels (Invitrogen)^45^.

### Protein digestion and TMT labelling

We performed the analysis with a previously optimized protocol^45,46^. For whole-proteome profiling, quantified protein samples (300 µg in the lysis buffer with 8 M urea) for each TMT channel were proteolysed with Lys-C (Wako, 1:100 w/w) at 21 °C for 2 h, and diluted by fourfold to reduce urea to 2 M for the addition of trypsin (Promega, 1:50 w/w) to continue the digestion at 21 °C overnight. The insoluble debris was kept in the lysates for the recovery of insoluble proteins. The digestion was terminated by the addition of 1% trifluoroacetic acid. After centrifugation, the supernatant was desalted with the Sep-Pak C18 cartridge (Waters), and then dried by Speedvac (Thermo Fisher). Each sample was resuspended in 50 mM HEPES (pH 8.5) for TMT labelling and then mixed equally, followed by desalting for the subsequent fractionation. For the whole-proteome analysis alone, 0.1 mg of protein per sample was used.

### Extensive two-dimensional liquid chromatography–tandem mass spectrometry

The TMT-labelled samples were fractionated by offline basic pH reverse phase liquid chromatography (LC), and each of these fractions was analysed by the acidic pH reverse phase liquid chromatography–tandem mass spectrometry (LC–MS/MS)^47,48^. We performed a 160-min offline LC run at a flow rate of 400 µl min^−1^ on an XBridge C18 column (3.5-μm particle size, 4.6 mm × 25 cm, Waters; buffer A: 10 mM ammonium formate, pH 8.0; buffer B: 95% acetonitrile, 10 mM ammonium formate, pH 8.0)^45^. A total of 80 2-min fractions were collected. Every 41st fraction was concatenated into 40 pooled fractions, which were subsequently used for whole-proteome TMT analysis.

In the acidic pH LC–MS/MS analysis, each fraction from basic pH LC was dried by a Speedvac and was run sequentially on a column (75 µm × 35 cm for the whole proteome, 50 µm × 30 cm for whole proteome, 1.9 µm of C18 resin from Dr. Maisch, 65 °C to reduce backpressure) interfaced with a Fusion mass spectrometer (Thermo Fisher) for the whole proteome where peptides were eluted by a 90 min gradient (buffer A: 0.2% formic acid, 5% DMSO; buffer B: buffer A plus 65% acetonitrile). Mass spectrometry (MS) settings included the MS1 scan (450–1600 *m*/*z*, 60,000 resolution, 1 × 10^6^ automatic gain control and 50-ms maximal ion time) and 20 data-dependent MS2 scans (fixed first mass of 120 *m*/*z*, 60,000 resolution, 1 × 10^5^ automatic gain control, 110-ms maximal ion time, higher-energy collisional dissociation, 36% normalized collision energy, 1.0 *m*/*z* isolation window with 0.2 *m*/*z* offset and 10-s dynamic exclusion).

### Protein identification and quantification with JUMP software

The computational processing of identification and quantification was performed with the JUMP search engine^47^. All original target protein sequences were reversed to generate a decoy database that was concatenated to the target database. Putative peptide spectrum matches (PSMs) were filtered by mass accuracy and then grouped by precursor ion charge state and filtered by JUMP-based matching scores (Jscore and ΔJn) to reduce false discovery rate (FDR) below 1% for proteins during the whole-proteome analysis. If one peptide could be generated from multiple homologous proteins, on the basis of the rule of parsimony, the peptide was assigned to the canonical protein form in the manually curated SwissProt database. If no canonical form was defined, the peptide was assigned to the protein with the highest PSM number. We performed the analysis in the following steps, as previously reported, with modifications^49^: (1) extracting TMT reporter ion intensities of each PSM; (2) correcting the raw intensities on the basis of the isotopic distribution of each labelling reagent (for example, TMT126 generates 91.8%, 7.9% and 0.3% of 126, 127 and 128 *m*/*z* ions, respectively); (3) excluding PSMs of very low intensities (for example, minimum intensity of 1,000 and median intensity of 5,000); (4) removing sample loading bias by normalization with the trimmed median intensity of all PSMs; (5) calculating the mean-centred intensities across samples (for example, relative intensities between each sample and the mean); (6) summarizing protein or phosphopeptide relative intensities by averaging related PSMs; (7) finally, deriving protein or phosphopeptide absolute intensities by multiplying the relative intensities by the grand-mean of three most highly abundant PSMs. In addition, we also performed *y*_1_ ion-based correction of TMT data. See Supplementary Data 1.

### Analysis of differentially expressed proteins

Differentially expressed proteins were identified using an empirical Bayes-moderated *t*-test to compare treatment groups with the limma R package (v.3.54.2)^50^. Low expressions were defined as the lower 25th percentile of the means of the protein expression, and proteins with a prevalence of low expression in more than 70% of the samples were filtered out. As a result, 7,731 out of 11,428 proteins were retained for further analysis. Criteria for differential expression included a *P* value < 0.01 and a fold-change greater than 1.5. Related volcano plots were created using the R package ggplot2 (v.3.5.0). The R environment used was v.4.3.2. PPI networks were constructed using STRINGdb (v.12)^51^, with a confidence threshold greater than 0.7. The resulting networks were imported and visualized using Cytoscape (v.3.5.10). Interaction data were sourced from text mining, experiments and existing databases. See Supplementary Data 1 and 2.

### Cell culture

HEK293T cells (Thermo Fisher) were a gift from B. E. Bernstein (Massachusetts General Hospital). Gesicle Producer 293T cells were a gift from D. R. Liu (Harvard University/Broad Institute) (Takara, catalogue no. 632617). K562 and CHLA-01-MED cells were obtained from ATCC. All mammalian cell lines were cultured in a humidified 5% CO_2_ incubator at 37 °C and routinely tested for mycoplasma (Sigma-Aldrich). ICB1299, CHLA-01-MED, and MED411FH-TC cells were cultured in stem cell media (50% DMEM/Nutrient Mixture F12 (DMEM/F12) plus 50% Neurobasal-A Medium supplemented with B-27 supplement (without vitamin A), 1 × GlutaMAX (Invitrogen), 1 mmol l^−1^ sodium pyruvate (Invitrogen), 1 × MEM Non-Essential Amino Acids Solution (Invitrogen), 25 mmol l^−1^ HEPES, 20 ng ml^−1^ basic fibroblast growth factor and 20 ng ml^−1^ epidermal growth factor). ICB1299 cells were cultured in Matrigel-coated plates and CHLA-01-MED and MED411FH-TC cells were cultured in low-attachment plates. HEK293F cells were obtained from Thermo Fisher. RPMI1640 and DMEM were supplemented with 100 U ml^−1^ penicillin and 100 µg ml^−1^ streptomycin (Gibco) and FBS (Peak Serum). K562 cells were cultured in RPMI1640 (Gibco) supplemented with 10% FBS. HEK293T and Gesicle Producer 293T cells were cultured in DMEM (Gibco) supplemented with 10% FBS. HEK293F cells were cultured in Freestyle 293 Expression Medium (Thermo Fisher) with shaking at 125 rpm. *Spodoptera frugiperda* (Sf9) insect cells (Expression Systems, catalogue no. 94-001F) were cultured in ESF921 media (Expression Systems) in a non-humidified and non-CO_2_ incubator at 27 °C with shaking at 140 rpm. High Five and ExpiSf9 cells were purchased from Thermo Fisher (catalogue nos. B85502 and A35243, respectively), with Grace insect medium (Thermo Fisher, catalogue no. 11595030) supplemented with 10% FBS (Cytiva) and 1% penicillin-streptomycin (Gibco), and cultured at 26 °C. All commercial cell lines were authenticated by short tandem repeat profiling (Genetica) and all cell lines were routinely tested for mycoplasma (Sigma-Aldrich).

### Lentiviral production

For lentivirus production, transfer plasmids were co-transfected with GAG/POL and VSV-G plasmids into 293T cells using Lipofectamine 3000 (Thermo Fisher) according to the manufacturer’s protocol. Medium was exchanged after 6 h and the viral supernatant was collected 52 h after transfection and sterile-filtered (0.45 µm). K562 cells were transduced by spinfection at 1,800*g* for 1.5 h at 37 °C with 8 µg ml^−1^ polybrene (Santa Cruz). Where necessary, 48 h after transduction, cells were selected with 600 µg ml^−1^ G418 sulphate (Thermo Fisher).

### Plasmid construction

Plasmids were cloned by Gibson Assembly using NEBuilder HiFi (New England Biolabs). Cloning strains used were NEB Stable (lentiviral) (New England Biolabs). Final constructs were validated by Sanger sequencing (Azenta/Genewiz).

All KBTBD4 expression plasmids encoded isoform 1 (human, residues 1–518) but longer isoform 2 (residues 1–534) numbering was used. CoREST expression plasmids encoded isoform 1 (human) full length (considered residues 4–485). Open reading frames (ORFs) of human KBTBD4 and CoREST (mammalian expression) were amplified from ORFs obtained from Horizon Discovery. The LSD1 ORF was a gift from R. Shiekhattar (University of Miami Miller School of Medicine). Full length HDAC1 ORF was a gift from E. Verdin (Addgene, catalogue no. 13820). The coding sequence of HDAC2 (amino acids 2–488) was synthesized by IDT. The coding sequence of full length NUDCD3 (human, residues 1–361) was synthesized by Twist Biosciences.

For transfection constructs, CoREST–FLAG and HA–KBTBD4 (WT or mutant) constructs were cloned into pcDNA3. For KBTBD4 overexpression constructs, KBTBD4 coding sequences were cloned into pSMAL mCherry, which was generated from pSMAL through introduction of an mCherry ORF into pSMAL (a gift from J. E. Dick, University of Toronto). For bacmid expression, KBTBD4 and NUDCD3 were cloned into pFastbac, a gift from T. Cech. For inducible expression constructs, KBTBD4 coding DNA sequence (CDS) was cloned into pInducer20 (Addgene, catalogue no. 44012). For eVLP constructs, sgRNA sequences were cloned into pU6-sgRNA (a gift from D. R. Liu, Harvard University/Broad Institute) by PCR amplification and co-transfected with pCMV-MMLVgag-3xNES-ABE8e (Addgene, catalogue no. 181751), pBS-CMV-gagpol (Addgene, catalogue no. 35614) and pCMV-VSV-G (Addgene, catalogue no. 8454), gifts from D. R. Liu, P. Salmon and B. Weinberg, respectively. eVLP sgRNA sequences are provided in Supplementary Table 1.

### Production of eVLPs

eVLPs were produced as previously described^22^. In brief, Gesicle Producer 293T cells were seeded in T-75 flasks (Corning) at a density of 5 × 10^6^ cells per flask. After 20–24 h, a mixture of plasmids expressing VSV-G (400 ng), MMLVgag–pro–pol (3,375 ng), MMLVgag–3xNES–ABE8e (1,125 ng) and an sgRNA (4,400 ng) were co-transfected into each T-75 flask using jetPRIME transfection reagent (Polyplus) according to the manufacturer’s protocols. At 40–48 h after transfection, producer cell supernatant was collected and centrifuged for 10 min at 4 °C and 2,000*g* to remove cell debris. The clarified eVLP-containing supernatant was filtered through a 0.45 μm PVDF filter (Sigma-Aldrich). The filtered supernatant was concentrated by ultracentrifugation using a cushion of 20% (w/v) sucrose (Sigma-Aldrich) in PBS. Ultracentrifugation was performed at 26,000 rpm for 2 h at 4 °C using an SW28 rotor in an Optima XE-90 Ultracentrifuge (Beckman Coulter). After ultracentrifugation, eVLP pellets were resuspended in cold PBS (pH 7.4). eVLPs were frozen and stored at −80 °C. eVLPs were thawed on ice immediately before use and repeated freeze–thaw was avoided.

### eVLP transduction in cell culture

K562 cells were plated for transduction in 96-well plates (Cellstar Greiner Bio-one) at a density of 50,000 cells per well with 5 µg ml^−1^ polybrene (Santa Cruz) media. Base editor (BE)-eVLPs were added directly to the culture media in each well. Next, 50 µl of fresh medium was added after 6 h, and another 100 µl of media was added 48 h after transduction. Then, 72 h after transduction, cellular genomic DNA was isolated and genotyped as described below. Transduced cells were allowed to recover for 7–10 days before degradation assays were performed.

For cell viability assays, ICB1299, CHLA-01-MED and MED411FH-TC were transduced with eVLPs and cultured in Stem cell media. Cells were collected on day 3 for genotyping. Cell viability was measured on day 4 (reference) and day 11 (end point) for ICB1299 and CHLA-01-MED or on day 3 (reference) and day 10 (end point) for MED411FH-TC using Cell Titer-Glo Luminescent Cell Viability Assay 2.0 (Promega) with PHERAstar FSX microplate reader. End point readings were normalized to that of reference to determine relative growth during 7 days of culture. For immunoblotting, ICB1299 cells were transduced with eVLPs and cultured in Stem cell media for 5 days before collecting for immunoblotting or genotyping. Primers used for genotyping are provided in Supplementary Table 2.

### Genotyping

Genomic DNA was extracted using QuickExtract DNA Extraction Solution (Biosearch Technologies) according to the manufacturer’s protocol. We subjected 100 ng of DNA to a first round of PCR (25–28 cycles, Q5 hot start high-fidelity DNA polymerase (New England Biolabs)) to amplify the locus of interest and attach common overhangs. Then, 1 µl of each PCR product was amplified in a second round of PCR (8 cycles) to attach barcoded adapters. Primer sequences are provided in Supplementary Tables 2 and 3. Final amplicons were purified by gel extraction (Zymo) and sequenced on an Illumina MiSeq. Data were processed using CRISPResso2 (ref. ^52^) using the following parameters: --quantification_window_size 20 --quantification_window_center -10 --plot_window_size 20 --exclude_bp_from_left 0 --exclude_bp_from_right 0 --min_average_read_quality 30 --n_processes 12 --base_editor_output.

### CRISPR–Cas9-mediated genome editing

#### Knock-in of CoREST–GFP in K562 cells

mEGFP followed by a ‘GGGSGGGS’ linker was knocked into the C terminus of CoREST (that is, *RCOR1*) in K562 cells. sgRNA (sgRNA: TTCAAAGCCACCAGTTTCTC) targeting the C terminus of CoREST was cloned into a Cas9 plasmid, PX459 (ref. ^53^), and electroporated according to the manufacturer’s protocol (Neon Transfection System, Thermo Fisher) with a repair vector containing the mEGFP CDS and linker flanked by 750 base pairs of genomic homology sequences to either side of the CoREST C terminus. In brief, 2 × 10^5^ cells were washed twice with PBS and resuspended in buffer R. PX459 (0.5 µg) and the repair vector (0.5 µg) were added to the cell suspension, and electroporated at 1,350 V with 10-ms pulse width for 4 pulses using the Neon Transfection System 10 µl kit. After electroporation, cells were immediately transferred to prewarmed media. To generate single-cell clones, cells were gated to sort for the top 0.2% GFP^+^ and single-cell sorted on a MoFlo Astrios EQ Cell Sorter (Beckman Coulter), and expanded and validated by western blot and Sanger sequencing.

#### Knock-in of HDAC2-dTAG in *HDAC1*-null CoREST–GFP K562 cells

Homology directed repair was used to insert a linker-FKBP12^F36V^-2xHA-P2A-Puro^R^ cassette into the C terminus of HDAC2 in *HDAC1*-null CoREST–GFP K562 cells (generation described below). sgRNA (sgRNA: GGTGAGACTGTCAAATTCAG) (Synthego) targeting the C terminus of HDAC2 was electroporated according to the manufacturer’s protocol (Neon Transfection System, Thermo Fisher) with a repair vector containing the linker-FKBP12^F36V^-2xHA-P2A-Puro^R^ CDS flanked by 700–800 base pairs of genomic homology sequences to either side of the HDAC2 C terminus. In brief, 2 × 10^6^ cells were washed twice with PBS and resuspended in buffer R. The sgRNA and the repair vector (0.5 µg) were added to the cell suspension, and electroporated at 1,350 V with 10-ms pulse width for 3 pulses using the Neon Transfection System 100 µl kit. After electroporation, cells were immediately transferred to prewarmed media. After 9 days of recovery, cells were selected with 2 µg ml^−1^ puromycin (Thermo Fisher) for 10 days before single-cell sorting on a MoFlo Astrios EQ Cell Sorter (Beckman Coulter). Single-cell clones were validated by Sanger sequencing and western blot.

#### Generation of knockout K562s

*HDAC1*-null, *HDAC2*-null and *KBTBD4*-null CoREST–GFP K562 clones were generated using the Alt-R CRISPR–Cas9 System (IDT) to deliver ribonucleoprotein complexes containing knockout (KO) guides (HDAC1: GCACCGGGCAACGTTACGAA; HDAC2: TACAACAGATCGTGTAATGA; KBTBD4: GATATCTGTGAGTAAGCGGT) using the Neon Transfection System (Thermo Fisher) according to the manufacturer’s protocol. Transfected cells were recovered for 72 h before sorting for single-cell clones on a MoFlo Astrios Cell Sorter (Beckman Coulter). Single-cell clones were validated by genotyping and immunoblotting. For LSD1 knockout, lentiviral vectors carrying sgRNA (LSD1) were generated by cloning appropriate sequences (LSD1: TAGGGCAAGCTACCTTGTTA) into pLentiCRISPR.v2 lentiviral vector. Control vector contained sgRNA targeting luciferase (sgControl). Lentivirus was produced and K562 CoREST–GFP cells were transduced and puromycin selected as described above. Primers and guide sequences used for genotyping are provided in Supplementary Tables 3 and 4, respectively.

### Degradation assay of KBTBD4 mutants

K562 *KBTBD4*-null CoREST–GFP cells were generated as described above. KBTBD4 overexpression constructs were cloned into pSMAL mCherry and point mutations were introduced into coding regions using standard PCR-based site-directed mutagenesis techniques. Lentiviral particles carrying the overexpression constructs were produced and used to transduce K562 *KBTBD4*-null CoREST–GFP cells as described above. At 48 h after transduction, GFP^+^ percentage was measured for mCherry^+^ cells in each condition (Supplementary Fig. 1a).

### Inducible expression of KBTBD4 mutants

Lentiviral particles carrying the inducible constructs were produced and used to transduce K562 cells as described above. At 48 h after transduction, cells were selected with 600 µg ml^−1^ G418 for 8–10 days. The selected cells were then treated with 1 µg ml^−1^ dox for indicated times with or without pre-treatment of DMSO, MLN4924 (1 µM), SAHA (10 µM), CI-994 (10 µM) or RBC1HI (10 µM). GFP^+^ percentage was measured for cells in each condition as shown in Supplementary Fig. 1a.

### Immunoblotting

Cells were lysed on ice in RIPA buffer (Boston BioProducts) with 1X Halt Protease Inhibitor Cocktail (Thermo Fisher) and 5 mM EDTA (Thermo Fisher). Lysate was clarified by centrifugation and total protein concentration was measured with the BCA Protein Assay (Thermo Fisher). Samples were electrophoresed and transferred to a 0.45-μm nitrocellulose membrane (Bio-Rad). Membranes were blocked with Tris-buffered saline Tween (TBST) with 5% Blotting-Grade Blocker (Bio-Rad) and incubated with primary antibody at the following dilutions: KBTBD4 (Novus Biologicals, catalogue no. NBP1-88587, 1:1,000), HDAC1 (Cell Signaling Technology, catalogue no. 34589, D5C6U, 1:1,000), HDAC2 (Cell Signaling Technology, catalogue no. 57156, D6S5P, 1:1,000), FLAG (Sigma-Aldrich, catalogue no. F1804, M2, 1:2,000), HA-tag (Cell Signaling Technology, catalogue no. 3724, C29F4, 1:1,000), GAPDH (Santa Cruz, catalogue no. sc-47724, 0411, 1:10,000). Membranes were washed three times with TBST and incubated with secondary antibody at the following dilutions: anti-rabbit IgG HRP conjugate (Promega, catalogue no. W4011, 1:20,000), anti-mouse IgG HRP conjugate (Promega, catalogue no. W4021, 1:40,000). Unless otherwise stated, following three washes with TBST, immunoblots were visualized using SuperSignal West Pico PLUS or SuperSignal West Femto chemiluminescent substrates (Thermo Fisher).

### Co-immunoprecipitation

HEK293T cells were transfected with 2 µg of pcDNA3 HA-KBTBD4 plasmid (mutant or WT) and with or without 3 µg of pcDNA3 CoREST–FLAG (full length or truncated) using PEI MAX transfection reagent (Polysciences) according to the manufacturer’s protocol. At 48 h after transfection, cells were treated with 1 µM MLN4924 for 3 h then with 1 µM UM171, 10 µM SAHA or vehicle for 1 h, or with 10 µM CI-994 for 3 h. Cells were washed twice with cold PBS and flash-frozen. Cells were thawed and lysed on ice in lysis buffer (25 mM Tris-HCl pH 7.5, 150 mM NaCl, 1% NP-40 alternative) supplemented with cOmplete, EDTA-free Protease Inhibitor Cocktail (Sigma-Aldrich), and the lysates were cleared. The protein concentration was quantified as above and diluted to 1 mg ml^−1^ in lysis buffer with 1 µM UM171 or DMSO. Supernatants were immunoprecipitated overnight at 4 °C with 25 µl of Pierce anti-HA magnetic beads (Thermo Fisher). Beads were washed six times with lysis buffer, eluted in SDS–PAGE loading buffer and carried forward to immunoblotting as described above.

### Protein expression and purifications

Human recombinant KBTBD4 for biochemical and biophysical analyses was purified from Sf9 insect cells. Complementary DNAs for human KBTBD4 and NUDCD3 proteins were cloned into the pFastBac donor vector and the recombinant baculoviruses were constructed using the Bac-to-Bac protocol and reagents (Thermo Fisher). KBTBD4 MB mutations were introduced into coding regions using standard PCR-based site-directed mutagenesis techniques. All KBTBD4 constructs were tagged on the N terminus with 6×His cleavable by TEV protease. These plasmids were used to prepare separate baculoviruses according to standard protocols (Bac-to-Bac Baculovirus Expression System, Thermo Fisher). Detection of gp64 was used to determine baculovirus titre (Expression Systems). For expression, SF9 cells were grown to a density of 1–2 × 10^6^ cells per millilitre and co-infected with NUDCD3 baculovirus at a multiplicity of infection of 2 and KBTBD4 baculovirus at a multiplicity of infection of 3.5. The cells were incubated for 72 h (27 °C, 120*g*), collected and then frozen with liquid nitrogen for future purification. Cells were resuspended in lysis buffer (50 mM Tris-HCl, pH 8.0 cold, 500 mM NaCl, 1 mM Tris(2-carboxyethyl)phosphine (TCEP), 10% glycerol, 15 mM imidazole) supplemented with 1% NP-40, 1 mM PMSF and Roche Complete Protease Inhibitor and sonicated. Lysate was clarified by centrifugation at 100,000*g* for 30 min and incubated with His60 Ni Superflow affinity resin (Takara). Resin was washed with lysis buffer containing a stepwise gradient of 15–50 mM imidazole, followed by elution using lysis buffer with 250 mM imidazole. Eluate was exchanged into storage buffer (50 mM Tris-HCl, pH 8.0 cold, 150 mM NaCl, 1 mM TCEP, 10% glycerol) using an Econo-Pac 10DG desalting column (Bio-Rad) and further purified by size exclusion chromatography using a Superdex 200 10/300 GL column (GE Healthcare). The purity of the recombinant protein was verified by SDS–PAGE and fractions with 90–95% purity were pooled and stored at −80 °C.

Recombinant human KBTBD4 used in cryo-EM structure determination was purified from *Trichoplusia ni* High Five insect cells. cDNAs for human KBTBD4 and NUDCD3 proteins were cloned into the pFastBac donor vector and the recombinant baculoviruses were constructed using the Bac-to-Bac protocol and reagents (Thermo Fisher). KBTBD4 constructs were tagged on the N terminus with 10×His and MBP tag cleavable by TEV protease. These plasmids were used to prepare separate baculoviruses according to standard protocols (Bac-to-Bac Baculovirus Expression System, Thermo Fisher). For expression, the monolayer High Five cells were grown to about 80% confluency and co-infected with NUDCD3 baculovirus. The cells were incubated for 72 h (26 °C), collected and then frozen with liquid nitrogen for future purification. Cells were resuspended in lysis buffer (50 mM Tris-HCl, pH 8.0 cold, 150 mM NaCl, 1 mM TCEP, 20 mM imidazole) supplemented with 1 mM PMSF, 10 µM leupeptin, 0.5 µM aproptinin and 1 µM pepstatin A and sonicated. Lysate was clarified by centrifugation at 100,000*g* for 30 min and incubated with amylose affinity resin (New England BioLabs). Resin was washed with lysis buffer, followed by elution using lysis buffer with 10 mM maltose. Eluate was cut with tobacco etch virus protease overnight, followed by the prepacked anion exchange column (GE Healthcare) to get rid of the protease, and further purified by size exclusion chromatography using a Superdex 200 10/300 GL column (GE Healthcare). The purity of the recombinant protein was verified by SDS–PAGE and fractions with 90–95% purity were pooled and stored at −80 °C.

Recombinant LSD1–CoREST–HDAC complex was composed of full length LSD1 (UniProt ID: O60341) or LSD1 (Δ77–86), full length HDAC1 (UniProt ID: Q13547) and N-terminally truncated CoREST (amino acids 86–485) (UniProt ID: Q9UKL0) or N-terminal Cys CoREST^26^. The pcDNA3 vector was used to create plasmids encoding the different proteins. The CoREST constructs contained an N-terminal (His)10(Flag)3 tag followed by a TEV protease cleavage site. The constructs for ternary complex were co-transfected into suspension-grow HEK293F cells (Thermo Fisher) with polyethylenimine (Sigma) and collected after 48 h. Cells were resuspended in lysis buffer (50 mM HEPES, pH 7.5, 100 mM KCl, 5% glycerol, 0.3% Triton X-100, 1X Roche EDTA-free Complete Protease Inhibitor cocktail) and sonicated. Lysate was clarified by centrifugation at 12,000*g* for 30 min and incubated with Anti-FLAG M2 affinity gel (Sigma). The affinity gel was washed twice with lysis buffer and twice with SEC buffer (50 mM HEPES, pH 7.5, 50 mM KCl, 0.5 mM TCEP) followed by the incubation with TEV protease overnight at 4 °C. The complex was further purified by size exclusion chromatography using a Superose 6 10/300 column (GE Healthcare). The purity of the complex was verified by SDS–PAGE and fractions with 90–95% purity were pooled and supplemented with 5% glycerol and stored at −80 °C.

Recombinant HDAC2–CoREST complex, composed of HDAC2 (amino acids 2–488) (UniProt ID: Q92769) and CoREST (amino acids 86–485), was purified from ExpiSf9 cells (Thermo Fisher). cDNAs for human HDAC2 and CoREST proteins were cloned into the pFastBac donor vector and the recombinant baculoviruses were constructed using the Bac-to-Bac protocol and reagents (Thermo Fisher). HDAC2 (amino acids 2–488) construct was tagged on the N terminus with SUMO tag, which can be cleaved in insect cells and with 6×His on the C terminus. CoREST (amino acids 86–485) was tagged with 10×His tag followed by an MBP tag on the N terminus. To improve the solubility of CoREST, six amino acids were mutated to the corresponding residues found in MIER2 (W172K F188C F191E V197A V201N F209K). These plasmids were used to prepare separate baculoviruses according to standard protocols (Bac-to-Bac Baculovirus Expression System, Thermo Fisher). For expression, the suspension ExpiSf9 cells were grown to about 5 × 10^6^ cells per millilitre and co-infected with HDAC2 and CoREST baculovirus. The cells were incubated for 72 h (26 °C), collected and then frozen with liquid nitrogen for future purification. Cells were resuspended in lysis buffer (50 mM Tris-HCl, pH 8.0 cold, 300 mM NaCl, 5 mM MaCl, 15% glycerol, 1 mM TCEP, 20 mM imidazole) supplemented with 1 mM PMSF, 10 µM leupeptin, 0.5 µM aproptinin and 1 µM epstatin A and sonicated. Lysate was clarified by centrifugation at 100,000*g* for 30 min and incubated with nickel affinity resin (Thermo Fisher). Resin was washed with lysis buffer, followed by elution using lysis buffer with 200 mM imidazole. Eluate was applied to the prepacked anion exchange column (GE Healthcare) to get rid of the contaminants and further purified by size exclusion chromatography using a Superdex 200 10/300 GL column (GE Healthcare). The purity of the recombinant protein was verified by SDS–PAGE and fractions with 90–95% purity were pooled and stored at −80 °C.

### Fluorescein labelling of LHC

The fluorescein labelling of the LSD1–CoREST–HDAC1 complex was purified as described above. A Cys point mutagenesis has been conducted next to the TEV protease cleavage site of N-terminally truncated CoREST for the ligation reaction with NHS-fluorescein^54^. A 2 mM NHS-fluorescein was incubated with 500 mM mercaptoethanesulfonate (MESNA) in the reaction buffer (100 mM HEPES, pH 7.5, 50 mM KCl, 1 mM TCEP) for 4 h at room temperature in the dark for transesterification. The LSD1–CoREST–HDAC1 complex purified by FLAG M2 affinity gel was washed with reaction buffer and incubated with TEV protease for 5 h at 4 °C. The complex was then mixed with 500 µl of the fluorescein/MESNA solution to make a final concentration of 0.5 mM fluorescein and 125 mM MESNA. The mixture was incubated for 48 h at 4 °C in the dark. The complex was desalted by a Zeba spin desalting column (7 kDa molecular weight cut-off) and further purified by size exclusion chromatography using a Superose 6 10/300 column (GE Healthcare). Fluorescein labelling efficiency was analysed by SDS–PAGE and fluorescence gel imaging (Amersham Typhoon FLA 9500, Cytiva). The purity of the complex was verified by SDS–PAGE and fractions with 90–95% purity were pooled and supplemented with 5% glycerol and stored at −80 °C.

### TR-FRET measurements

Unless otherwise noted, experiments were performed in white, 384-well microtitre plates (Corning, catalogue no. 3572) in 30-μl assay volume, or white, 384-well low-volume microtitre plates (PerkinElmer, catalogue no. 6008280). TR-FRET measurements were acquired on a Tecan SPARK plate reader with SPARKCONTROL software v.2.1 (Tecan Group), with the following settings: 340/50-nm excitation, 490/10-nm (Tb) and 520/10-nm (FITC, AF488) emission, 100-μs delay, 400-μs integration. The 490/10-nm and 520/10-nm emission channels were acquired with a 50% mirror and a dichroic 510 mirror, respectively, using independently optimized detector gain settings unless specified otherwise. The TR-FRET ratio was taken as the 520/490-nm intensity ratio on a per-well basis.

### Ternary complex measurements by TR-FRET

#### Titration of fluorescein-labelled LSD1–CoREST–HDAC complex

Recombinant WT (or mutant) 6×His–KBTBD4 (10 nM, 2×) and CoraFluor-1-labelled anti-6×His IgG (5 nM, 2×)^29^ were diluted into LHC buffer, with or without 10 μM UM171, and 5 μl added to wells of a white, 384-well low-volume microtitre plate (PerkinElmer, catalogue no. 6008280). Serial dilutions of fluorescein-labelled LSD1–CoREST–HDAC complex (1:2 titration, 10-point, *c*_max_ = 1,000 nM, 2×) were prepared in ligand buffer and 5 μl added to wells of the same plate (final volume 10 μl, final 6×His–KBTBD4 concentration 5 nM, final CoraFluor-1-labelled anti-6×His IgG concentration 2.5 nM, fluorescein-labelled LSD1–CoREST–HDAC complex *c*_max_ = 500 nM). The plate was allowed to equilibrate for 1 h at room temperature before TR-FRET measurements were taken. Data were background-corrected from wells containing no 6×His–KBTBD4. Prism 9 was used to fit the data to a four-parameter dose–response curve.

#### Titration of InsP_6_

Recombinant WT or mutant (P and PR) 6×His–KBTBD4 (40 nM), fluorescein-labelled LSD1–CoREST–HDAC complex (40 nM) and CoraFluor-1-labelled anti-6×His IgG (20 nM)^29^ were diluted into a one-to-one mixture of ligand buffer (50 mM Tris-HCl, pH 8.0, 150 mM NaCl, 1 mM TCEP, 10% glycerol) and LHC buffer (20 mM HEPES, pH 7.5, 1 mM TCEP, 2 mg ml^−1^ BSA, 0.1% Tween-20) and 10 μl added to wells of a white, 384-well low-volume microtitre plate (PerkinElmer, catalogue no. 6008280). InsP_6_ was added in serial dilution (1:10 titration, 6-point, *c*_max_ = 100 μM) using a D300 digital dispenser (Hewlett-Packard), and allowed to equilibrate for 1 h at room temperature before TR-FRET measurements were taken. Data were background-corrected from wells containing no InsP_6_. Prism 9 was used to fit the data to a four-parameter dose–response curve.

#### Incubation with HDAC inhibitor or UM171

Fluorescein-labelled LSD1–CoREST–HDAC complex (100 nM) and CoraFluor-1-labelled anti-6×His IgG (20 nM)^29^ were diluted into a one-to-one mixture of ligand buffer (50 mM Tris-HCl, pH 8.0, 150 mM NaCl, 1 mM TCEP, 10% glycerol) and LHC buffer (20 mM HEPES, pH 7.5, 1 mM TCEP, 2 mg ml^−1^ BSA, 0.1% Tween-20, 100 µM InsP_6_) and 10 μl added to wells of a white, 384-well low-volume microtitre plate (PerkinElmer, catalogue no. 6008280). HDACi (SAHA, CI-994, RBC1HI) (10 µM), UM171 (10 µM) or vehicle (DMSO) was added using a D300 digital dispenser (Hewlett-Packard), and allowed to equilibrate for 1 h at room temperature. Recombinant WT or mutant (P or PR) 6×His–KBTBD4 (100 nM) was then added using a D300 digital dispenser (Hewlett-Packard), and allowed to equilibrate for 1 h at room temperature before TR-FRET measurements were taken. Data were background-corrected from wells containing no 6×His–KBTBD4. Prism 9 was used plot the data.

### In vitro ubiquitination assay

The ubiquitination assays were set up similarly to as previously reported^55^. Reactions were performed at 37 °C in a total volume of 20 µl. The reaction mixtures contained 5 mM ATP, 100 μM WT ubiquitin, 100 nM E1 protein, 2 μM E2 protein, 0.5 μM neddylated RBX1-CUL3, 0.5 µM WT or PR KBTBD4 (unless otherwise indicated), with 25 mM Tris-HCl (pH 7.5), 20 mM NaCl, 10 µM InsP_6_ and 2.5 mM MgCl_2_ as reaction buffer. Substrate LHC at 0.5 µM was preincubated with everything except E1 in the reaction mixture at 37 °C for 5 min before adding E1 to initiate the reaction. Reactions were quenched at the indicated time points by adding SDS loading buffer containing reducing agent β-mercaptoethanol. The reaction samples were resolved on SDS–PAGE gels and analysed by Colloidal Blue staining, western blots or Typhoon fluorescent imaging.

### Deep mutational scan

The library of KBTBD4 mutants in the 7-amino acid region between Gly307 and Arg313 was designed to comprise all possible: (1) deletions, (2) 1-amino acid substitutions, (3) 2-amino acid substitutions of adjacent residues, (4) 1-amino acid insertions, (5) 2-amino acid insertions, (6) 3-amino acid insertions of GGG or GSG, and (7) 100 randomly scrambled WT sequences and the 2 remaining MB indels (PR311delinsPPHV, IPR310delinsTTYML). The 5′ and 3′ homology arms were added as well as forward and reverse barcodes for the different sub pools of mutations for downstream cloning. The final library was ordered from Twist Biosciences as a pooled oligo library with final lengths of single-stranded oligos ranging from 101 to 113 nucleotides (Supplementary Data 3). The Twist pool was resuspended in tris-EDTA to the concentration of 1 ng μl^−1^ and the sub pools were separated by PCR amplification of 22 cycles, using lsPCR1 primers listed in Supplementary Table 5 and using 1 ng of the Twist pool as template in each reaction. Each sub pool was further amplified with lsPCR2 primers in Supplementary Table 5 by PCR amplification of 10 cycles and the library pools were gel purified (Zymo Gel DNA Recovery Kit). Oligos corresponding to 2–6-amino acid deletions were ordered from Sigma-Aldrich and cloned separately from the Twist pool (Supplementary Data 3).

The mutational library was cloned into pSMAL mCherry using Gibson assembly. The backbone for the Gibson assembly was prepared by introducing a BamHI restriction site in place of residues Gly307 and Arg313 using primers in Supplementary Table 6. The backbone was digested with BamHI (NEB) and subsequently treated with Antarctic phosphatase (NEB) and the correct linearized backbone was isolated by gel electrophoresis and purified using Gel DNA Recovery Kit (Zymo). Then, 190 ng of linearized vector and 13.15 ng of each sub pool were used for each Gibson reaction of 80 µl using HIFI DNA Assembly Master Mix (NEB). The Gibson reaction was incubated for 1 h at 50 °C and DNA was isolated by isopropanol precipitation and transformed into Lucigen Endura Competent Cells according to the manufacturer’s protocol. Cells were recovered in Lucigen Endura Recovery Media for 1 h at 30 °C and later plated and grown overnight at 30 °C. Colonies were collected and the plasmid library was extracted using QIAGEN Plasmid Maxi Kit. Purified sub pools were then combined for the final library and sequence verified on an Illumina MiSeq as previously described.

Lentivirus was produced and titred by measuring cell counts after transduction and mCherry selection. K562 *KBTBD4*-null CoREST–GFP knock-in cells were transduced with library lentivirus at a multiplicity of infection less than 0.3 and, at day 3 after transduction, cells were sorted on a MoFlo Astrios Cell Sorter (Beckman Coulter), collecting the top 10% GFP^−^ and mCherry^+^, GFP^+^ and mCherry^+^, and mCherry^+^ (GPF^+/−^) cells. Genomic DNA was isolated using the QIAamp DNA Blood Mini kit or QIAamp UCP DNA Micro kit, and mutation sequences were amplified using barcoded primers listed in Supplementary Table 7, purified by gel extraction and sequenced on an Illumina MiSeq as previously described. Sorting was performed in three reps and, at all steps, greater than 150× coverage of the library was maintained.

We analysed data using Python (v.3.9.12) with Biopython (v.1.78), Pandas (v.1.5.1) and NumPy (v.1.23.4). In brief, raw reads matching sequences in the mutational library from unsorted as well as sorted (GFP^+^ and GFP^−^) cells were counted. Counts were then processed by converting them to reads per million, adding a pseudocount of 1 and transforming them by log_2_. Enrichment of each variant in GFP^+^ and GFP^−^ populations was quantified by subtracting the GFP^+^ and GFP^−^ log_2_-transformed counts, respectively, by corresponding log_2_-transformed counts for unsorted cells and averaged across replicates (Supplementary Data 4). Heatmaps were generated using matplotlib (v.3.7.1).

### Analysis of sequence motifs

Position probability matrices of the GFP^+^ and unsorted populations were constructed for each mutually exclusive category (single substitution, single insertion, double substitution and double insertion) by normalizing raw counts by the total read counts of each corresponding category, averaging across replicates and tallying the probability of every amino acid at each position. The information content, IC, of each position *N* was calculated according to Kullback–Leibler divergence, which is as follows:

$${\rm{IC}}(N)=P(N)\times {\log }_{2}\frac{P(N)}{{B}_{N}}$$

where *P*(*N*) is the position probability matrix of the GFP^+^ population for each mutational category, and the position probability matrix of the unsorted population was used as background frequencies *B*_*N*_. Logos were generated using Logomaker (v.0.8)^56^.

### Ex vivo drug sensitivity screening in MB PDX cells

MB PDXs harbouring WT KBTBD4 (RCMB28 *n* = 3, MED411FH *n* = 2) or KBTBD4-PR mutant (ICB1572 *n* = 5) were used to assess sensitivity to the HDAC1/2 inhibitor RBC1HI. In brief, freshly resected PDX tumours were cut into small pieces, incubated for 30 min at 37 °C in papain solution (10 units per millilitre, Worthington, catalogue no. LS003126) containing *N*-acetyl-l-cysteine (160 μg ml^−1^, Sigma-Aldrich, catalogue no. A9165) and DNase I (12 μg ml^−1^, Sigma-Aldrich, catalogue no. DN25) and dissociated to single cells by gentle pipetting. Red blood cells in the tumour cell suspension were removed by incubating in RBC Lysis buffer (STEMCELL technologies, catalogue no. 07850) at 37 °C for 2 min, followed by rinsing in DPBS-BSA. Cells were filtered using a 40-µm strainer and counted, and viability assessed to be above 80%. Cells were plated at 1,000 cells per well in 384-well plates in Stem cell media. Serially diluted RBC1HI was immediately added at a final concentration of 40–0.006 µM to the plated cells, with DMSO as negative control, and incubated for 72 h. Cell viability at the end of incubation was measured using Cell Titer-Glo Luminescent Cell Viability Assay 2.0 (Promega) with PHERAstar FSX microplate reader. Raw values were converted to cell viabilities and data analysed using Prism 10 to generate dose–response curves and obtain half-maximum inhibitory concentration values^57^.

### Ex vivo degradation assay in MB PDX cells

KBTBD4-PR mutant PDX (ICB1572) tumour was freshly isolated from mouse cerebellum, dissociated to a single-cell suspension and plated at 1 × 10^6^ cells per well in a six-well plate in Stem cell media. Cells were immediately dosed with the HDAC1/2 inhibitor RBC1HI or the NEDD8-activating enzyme inhibitor MLN4924 and incubated at 95% humidity and 5% CO_2_. Cells were collected 24 h later and lysed in RIPA buffer, and immunoblotting was performed. Immunoblot images were captured using the LICOR Odyssey CLX Imaging system.

### Cryo-EM sample preparation and data collection

To assemble the complexes of KBTBD4-PR/TTYML–LHC and KBTBD4-PR–HDAC2–CoREST for cryo-EM study, the individually isolated KBTBD4-mutant proteins and co-expressed LHC or HDAC2–CoREST complex were mixed in stoichiometric amounts with 100 mM InsP_6_ added and subsequently applied to the Superose 6 increase gel filtration column (Cytiva) in a buffer containing 40 mM HEPES, pH 7.5, 50 mM KCl, 100 mM InsP_6_ and 0.5 mM TCEP. The isolated complex was then crosslinked with 37.5 mM glutaraldehyde at room temperature for 6 min and the reaction quenched with 1 M Tris-HCl pH 8.0. The crosslinked sample was snap-frozen for future use.

To prepare grids for cryo-EM data collection, a QuantiFoil Au R0.6/1 grid (Electron Microscopy Sciences) was glow discharged for 30 s at 20 mA with a glow discharge cleaning system (PELCO easiGlow). Then, 3.0 μl of the purified and crosslinked KBTBD4-PR/TTYML–LHC complex at 0.7 mg ml^−1^ or KBTBD4-PR–HDAC2–CoREST complex at 0.5 mg ml^−1^ was applied to a freshly glow-discharged grid. After incubating in the chamber at 10 °C and 100% relative humidity, grids were blotted for 3 s with a blotting force of zero, then immediately plunge-frozen in liquid ethane using a Vitrobot Mark IV system (Thermo Fisher). Data collection of KBTBD4-PR–LHC and KBTBD4-PR–HDAC2–CoREST was carried out on an FEI Titan Glacios and Krios transmission electron microscope (Thermo Fisher) operated at 200 kV and 300 kV, respectively, at the Arnold and Mabel Beckman Cryo-EM Center of the University of Washington. An automation scheme was implemented using the SerialEM software using beam-image shift at a nominal magnification of 105 K, resulting in a physical pixel size of 0.84 Å. The images were acquired on a K3 camera direct detector. The dose rate was set to 10 e^−^ Å^−2^ s^−1^, and the total dose of 50 electrons per Å^2^ for each image was fractionated into 99 electron-event representation frames. Data collection of KBTBD4-TTYML–LHC was carried out on a Krios transmission electron microscope (Thermo Fisher) operated at 300 kV at the HHMI Janelia Research Campus. An automation scheme was implemented using the SerialEM^58^ software using beam-image shift^59^ at a nominal magnification of 165 K, resulting a physical pixel size of 0.743 Å. The images were acquired on a Falcon 4i camera direct detector, with the slit width of Selectris X (Thermo Fisher) set to be 6 eV. The dose rate was set to 15.39 e^−^ Å^−2^ s^−1^, and the total dose of 60 electrons per Å^2^ for each image was fractionated into 60 electron-event representation frames. Data were collected in four sessions with a defocus range of 0.8–1.5 μm. In total, 6,839 and 8,414 videos were collected for KBTBD4-PR–LHC and KBTBD4-TTYML–HC, respectively. For KBTBD4-PR–HDAC2–CoREST, data were collected in four sessions with a defocus range of 0.8–1.8 μm. In total, 11,263 videos were collected.

### Image processing and 3D reconstruction

For all three complexes, videos were collected and imported into CryoSPARC^60^ followed by patch motion correction and patch the contrast transfer function (CTF) estimation. Micrographs were kept after filtering the micrographs with CTF parameters and manual inspection. Blob picker job in CryoSPARC was able to pick particles, which were further extracted and subjected to two-dimensional classification. After five rounds of cleaning by two-dimensional classification, particles were selected and subjected to ab initio reconstruction. Subsequently, all particles were used for heterogenous refinement. After one extra round of cleaning up by heterogenous refinement, particles from good reconstruction were selected to get re-extracted without Fourier cropping. Homogenous refinement and non-uniform refinement^61^ help achieve an overall final resolution. To optimize the map for the KELCH-repeat domain, a soft mask focused on the KELCH domains was applied to local refinement, ending up with a further improved resolution. Topaz picker was used to pick more particles for a second round ab initio construction and refinements to achieve further resolution improvement. More details about the data processing can be found in Extended Data Figs. 5–7.

### Model building and refinement

The initial structural models of the KBTBD4 dimer, the HDAC1/2–CoREST–ELM–SANT1 complex, were predicted with AlphaFold-Multimer in Google ColabFold2 (ref. ^62^). The structural models of KBTBD4 BTB-BACK domain, KELCH-repeat domain and HDAC1–CoREST were separately fit into the cryo-EM map using UCSF ChimeraX-1.7 (rc2023.12.12)^63^. The resulting model was subsequently rebuilt in Coot (0.9.8.91)^64^ on the basis of the protein sequences and the electron microscopy density and was further improved by real-space refinement in PHENIX (1.20.1-4487-000)^65,66^. The structure figures were made using PyMOL^67^.

### Reporting summary

Further information on research design is available in the Nature Portfolio Reporting Summary linked to this article.

## Online content

Any methods, additional references, Nature Portfolio reporting summaries, source data, extended data, supplementary information, acknowledgements, peer review information; details of author contributions and competing interests; and statements of data and code availability are available at 10.1038/s41586-024-08533-3.

## Supplementary information

Supplementary Information This file contains Supplementary Figs. 1–7 and Tables 1–7.

Reporting Summary

Supplementary Data 1 KBTBD4 MUT versus WT PDX TMT proteomics data. Protein abundances identified from *KBTBD4*^*MUT*^ and *KBTBD4*^*WT*^ PDX models (source data for Fig. 1c,d and Extended Data Fig. 1d,e).

Supplementary Data 2 KBTBD4 MUT versus WT PDX proteomics data. Proteins identified from *KBTBD4*^*MUT*^ and *KBTBD4*^*WT*^ PDX models (shown in Fig. 1c,d and Extended Data Fig. 1d,e). An empirical Bayes-moderated *t*-test was used to compare treatment groups, using the limma R package.

Supplementary Data 3 Deep mutational scanning library sequences. Amino acid sequences provided begin at residue 307. Full sequences include mutational loop sequence, forward and reverse barcodes, and homologous sequences up- and downstream of the mutational loop sequence.

Supplementary Data 4 Deep mutational scanning enrichment scores. Source Data for deep mutational scanning (Figs. 2 and 3f,h and Extended Data Figs. 4, 5 and 10): raw read counts and (log_2_ + 1)-transformed mutant read-count-normalized reads.

## Source data

Source Data Fig. 1

Source Data Fig. 5

Source Data Extended Data Fig. 2

Source Data Extended Data Fig. 3

Source Data Extended Data Fig. 9

Source Data Extended Data Fig. 10

## Figures and Tables

**Extended Data Table 1:**
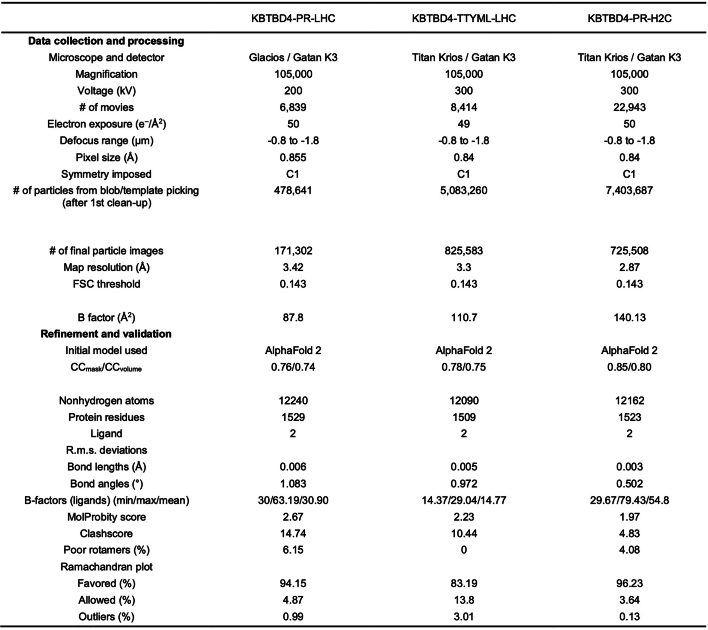
Cryo-EM data collection, refinement, and validation statistics

KBTBD4-PR-LHC, KBTBD4-TTYML-LHC, and KBTBD4-PR-H2C refer to the complex structures of the KBTBD4-PR mutant bound to LSD1-HDAC1-CoREST, the KBTBD4-TTYML mutant bound to LSD1-HDAC1-CoREST, and the KBTBD4-PR mutant bound to HDAC2-CoREST, respectively.

## Data Availability

The coordinates and density maps of the KBTBD4-PR–LHC–InsP_6_, KBTBD4-TTYML–LHC–InsP_6_ and KBTBD4-PR–HDAC2–CoREST complexes are deposited in the Protein Data Bank (PDB) with the accession numbers 8VRT, 8VPQ and 9DTQ, and in the Electron Microscopy Data Bank (EMDB) with the accession numbers EMD-43487, EMD-43413 and EMD-47156, respectively. DepMap (24Q4 release) was downloaded from https://depmap.org/portal/. The following publicly available dataset was used: PDB accession code 4LXZ. MS-based proteomics raw data files, DMS data and oligonucleotide sequences, as well as additional data generated by this study, are provided as Supplementary Information.Source data are provided with this paper.

## Code availability

Code generated for data analysis is available at https://github.com/liaulab/KBTBD4_DMS_2024.
