# Supplementary material for: Converging mechanism of UM171 and KBTBD4 neomorphic cancer mutations
Source: Nature. Author manuscript; Available in PMC 2025 Mar 13. (PMC11882451; doi:10.1038/s41586-024-08533-3)

nature portfolio

https://doi.org/10.1038/s41586-024-08533-3

Supplementary information

Converging mechanism of UM171 and KBTBD4 neomorphic cancer mutations

In the format provided by the authors and unedited

Supplementary Information for

Converging mechanism of UM171 and KBTBD4 neomorphic cancer mutations

Xiaowen Xie, Olivia Zhang, Megan J.R. Yeo, Ceejay Lee, Ran Tao, Stefan A. Harry, N. Connor Payne, Eunju Nam, Leena Paul, Yiran Li, Hui Si Kwok, Hanjie Jiang, Haibin Mao, Jennifer L. Hadley, Hong Lin, Melissa Batts, Pallavi M. Gosavi, Vincenzo D'Angiolella, Philip A. Cole, Ralph Mazitschek, Paul A. Northcott, Ning Zheng*, Brian B. Liau*

***Correspondence:** nzheng@uw.edu, liau@chemistry.harvard.edu

Supplementary Figures

**Supplementary Figure 1**	Representative gating schemes for flow cytometry	**2**
**Supplementary Figure 2**	Uncropped western blot images in Fig. 1	**3**
**Supplementary Figure 3**	Uncropped western blot images in Fig. 5	**4**
**Supplementary Figure 4**	Uncropped western blot images in ED Fig. 1	**5**
**Supplementary Figure 5**	Uncropped western blot images in ED Fig. 2	**6**
**Supplementary Figure 6**	Uncropped western blot and gel images in ED Fig. 3	**8**
**Supplementary Figure 7**	Uncropped western blot images in ED Fig. 10	**9**

Supplementary Tables

**Supplementary Table 1**	sgRNA sequences for eVLP production	**10**
**Supplementary Table 2**	Sequencing primers for base editing validation	**11**
**Supplementary Table 3**	Sequencing primers for knockout experiments	**12**
**Supplementary Table 4**	sgRNA sequences for knockout experiments	**13**
**Supplementary Table 5**	Primers used for DMS library cloning	**14**
**Supplementary Table 6**	Primers used for DMS plasmid construction	**15**
**Supplementary Table 7**	Primers used for DMS sequencing	**16**

Supplementary Methods

Synthetic Procedures and NMR Spectra **18**

Supplementary Figure 1 | Representative gating schemes for flow cytometry

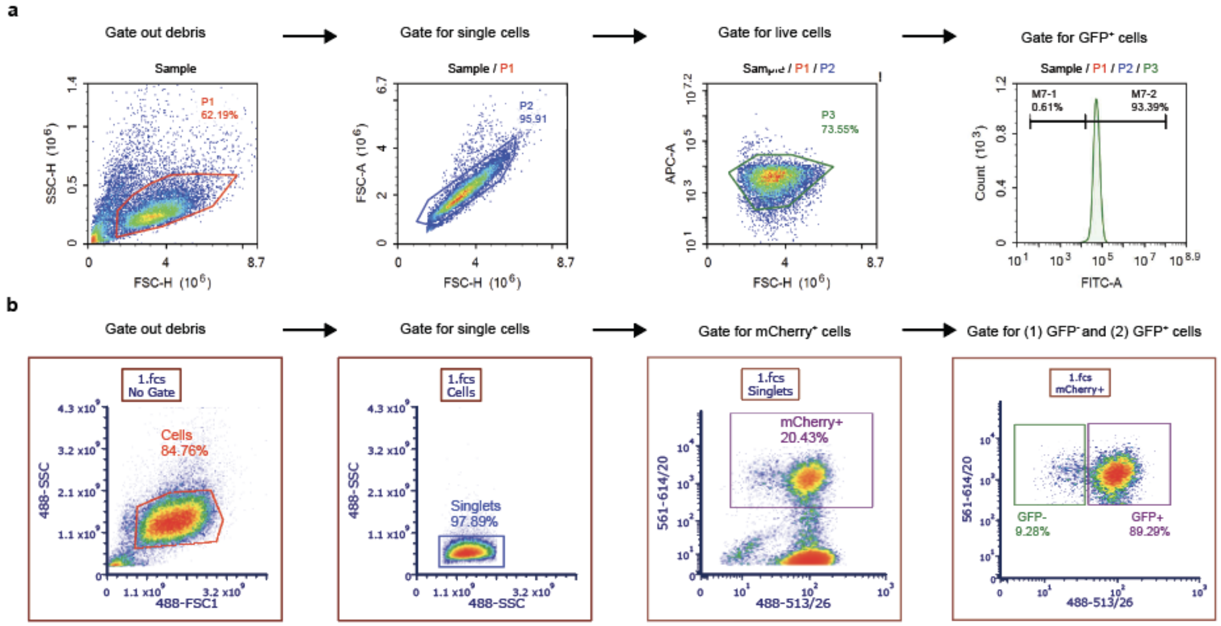

a, Representative gating scheme for flow cytometric analysis of CoREST-GFP degradation by KBTBD4. Helix NP NIR was used as a viability dye. GFP fluorescence was monitored on the FITC channels. **b**, Representative gating scheme for flow cytometric analysis of CoREST-GFP degradation by KBTBD4. GFP fluorescence and mCherry fluorescence were monitored on the FITC and PE-Texas Red channels, respectively. Data shown is from the deep mutational scanning.

Supplementary Figure 2 | Uncropped western blot images in Figure 1

Fig 1e

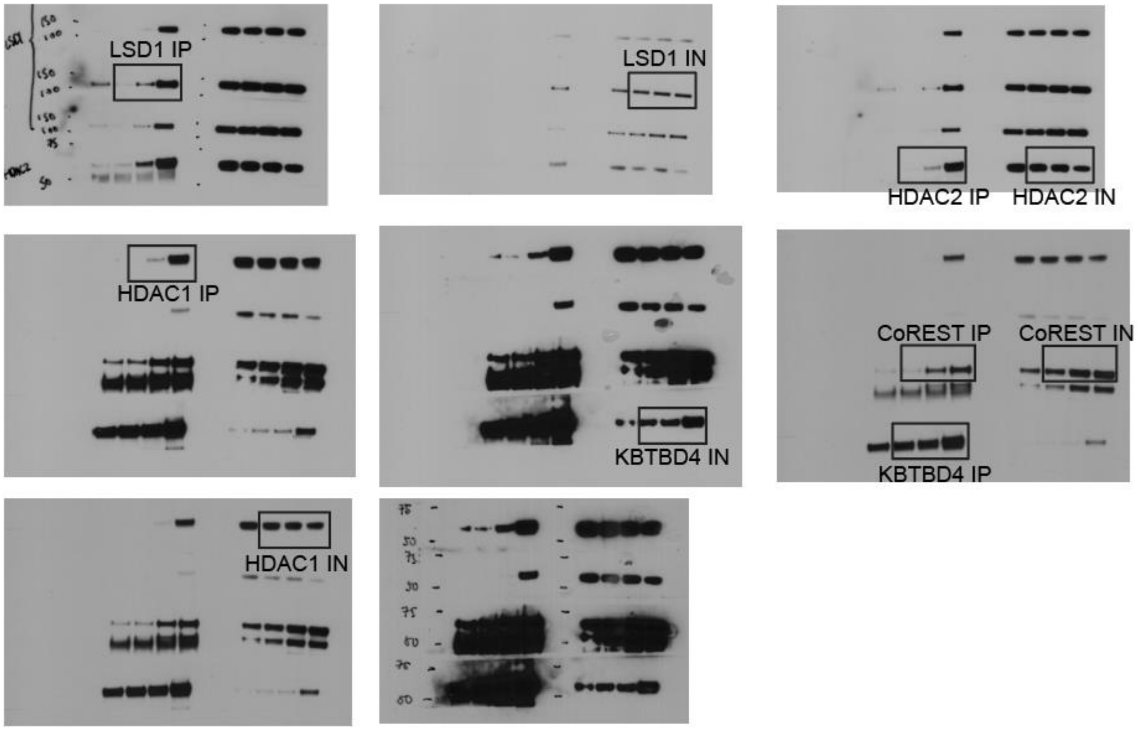

Fig 1f

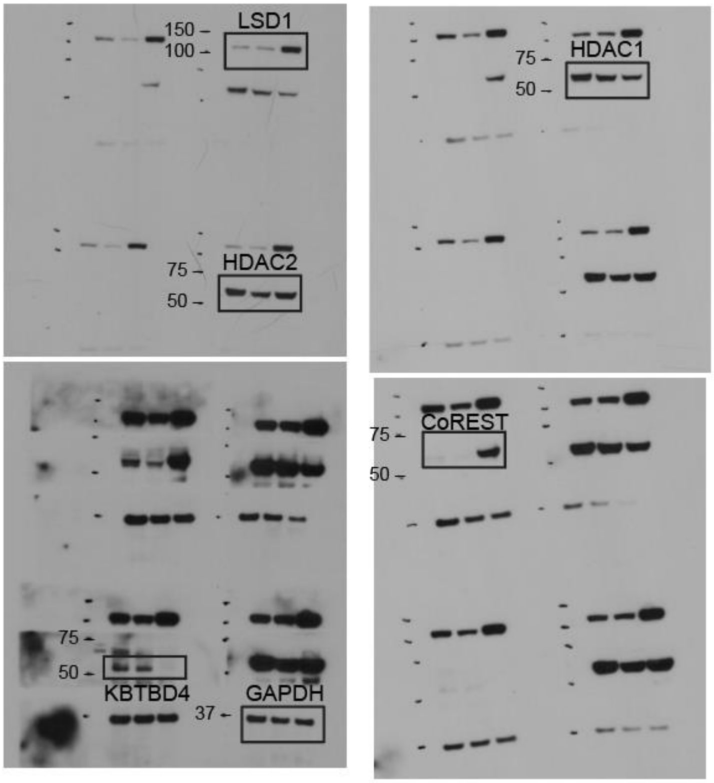

Fig 1k

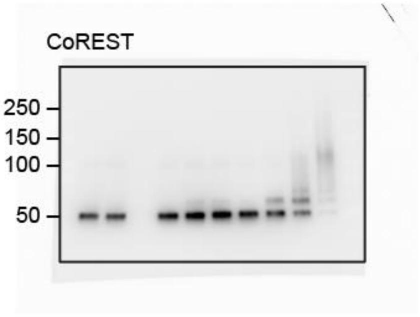

Supplementary Figure 3 | Uncropped western blot images in Figure 5

Fig 5c

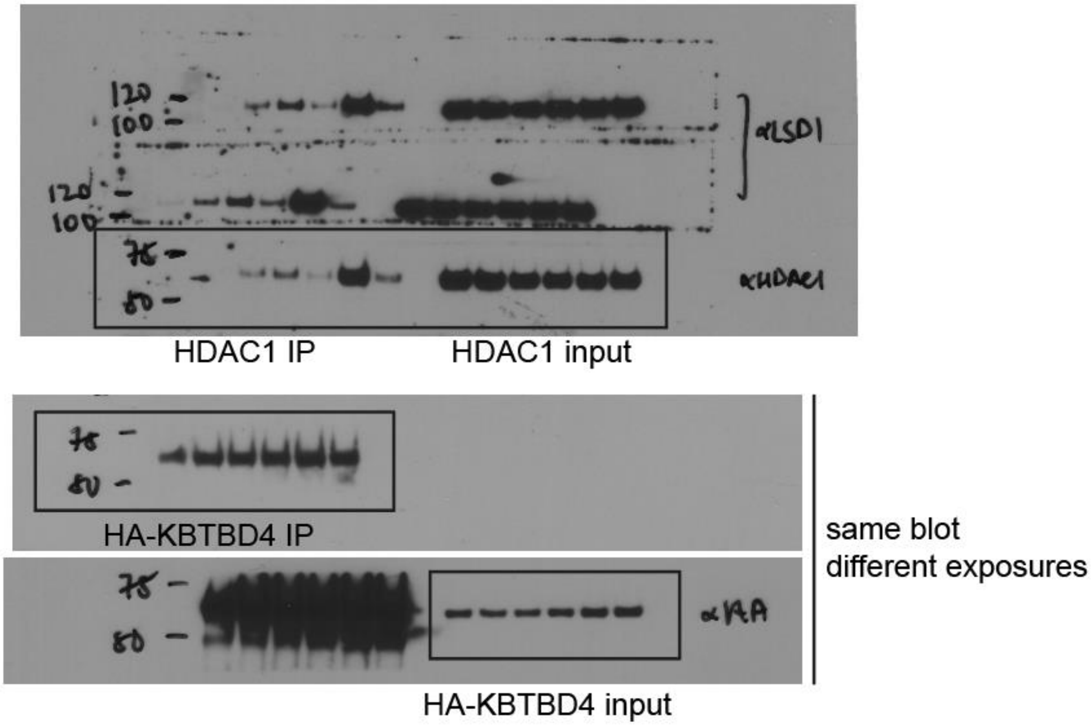

Fig 5f

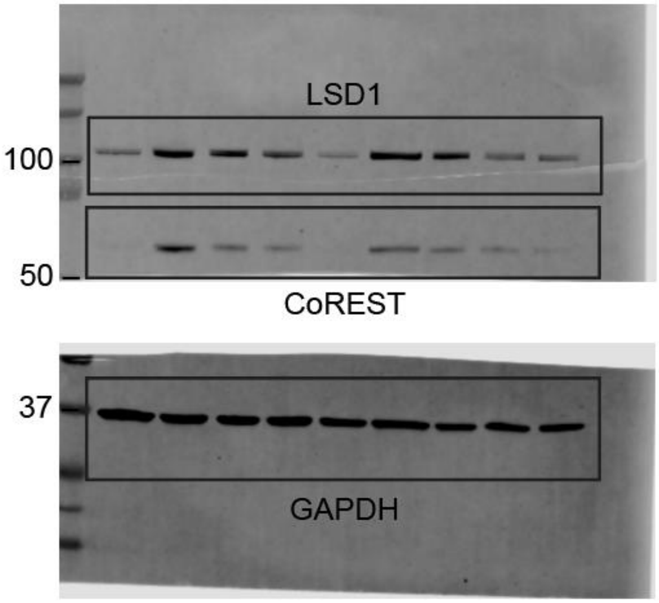

Supplementary Figure 4 | Uncropped western blot images in Extended Data Figure 1

ED Fig 1b - same blot different exposures

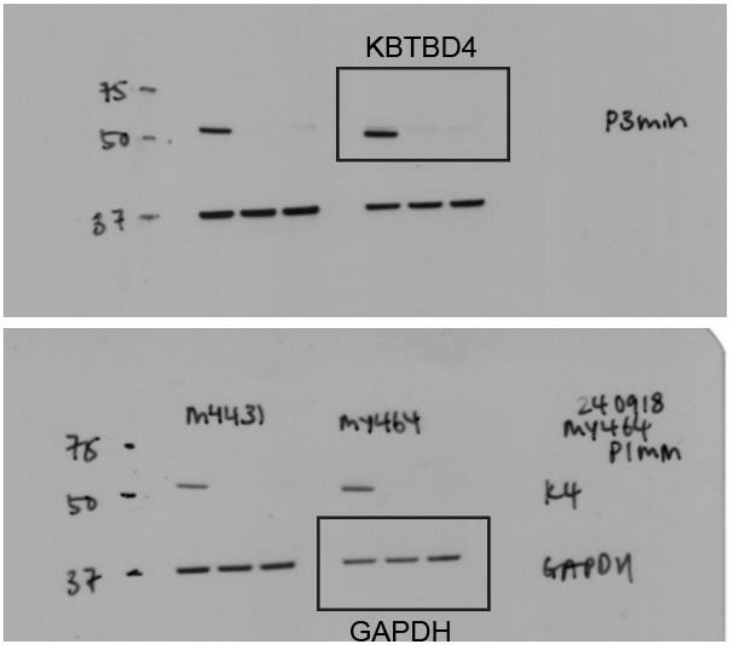

Supplementary Figure 5 | Uncropped western blot images in Extended Data Figure 2

ED Fig 2a

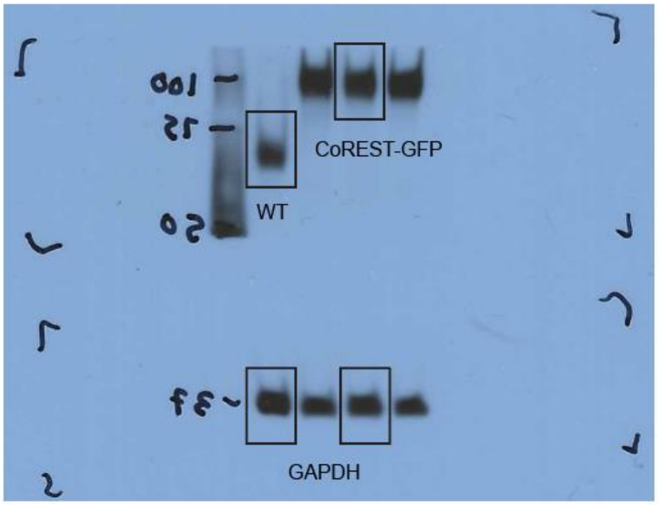

ED Fig 2b

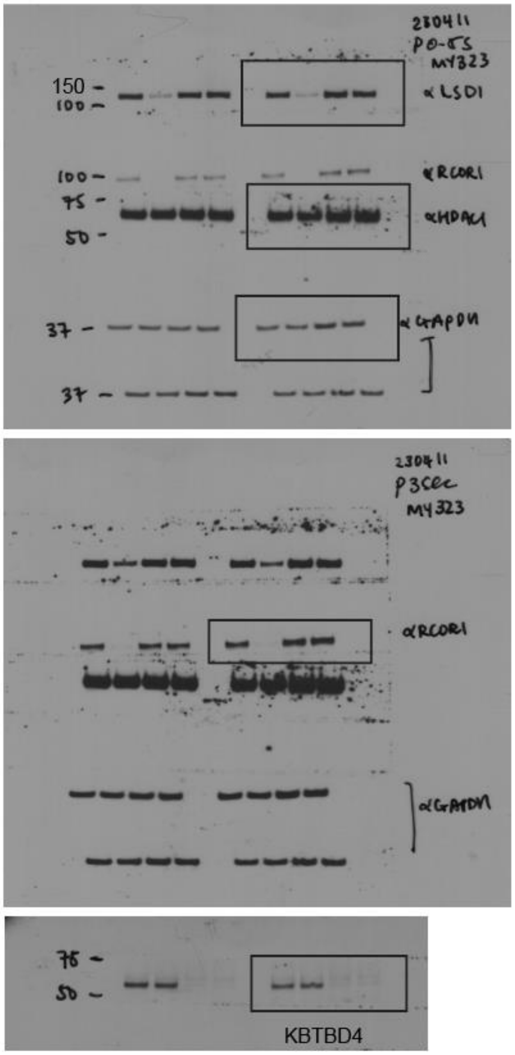

ED Fig 2c

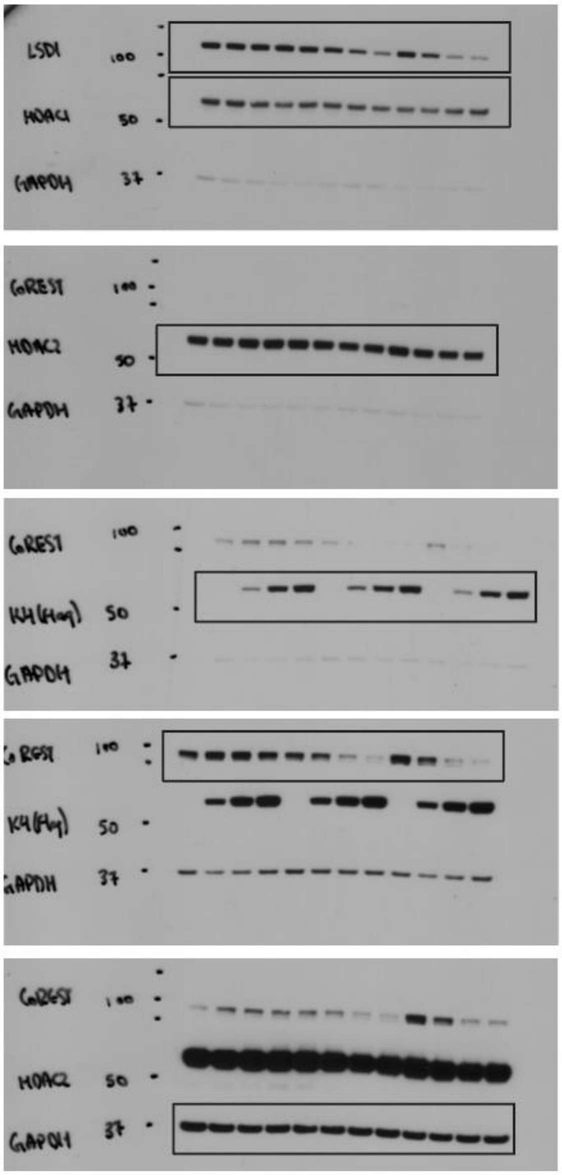

ED Fig 2e - same blots different exposure

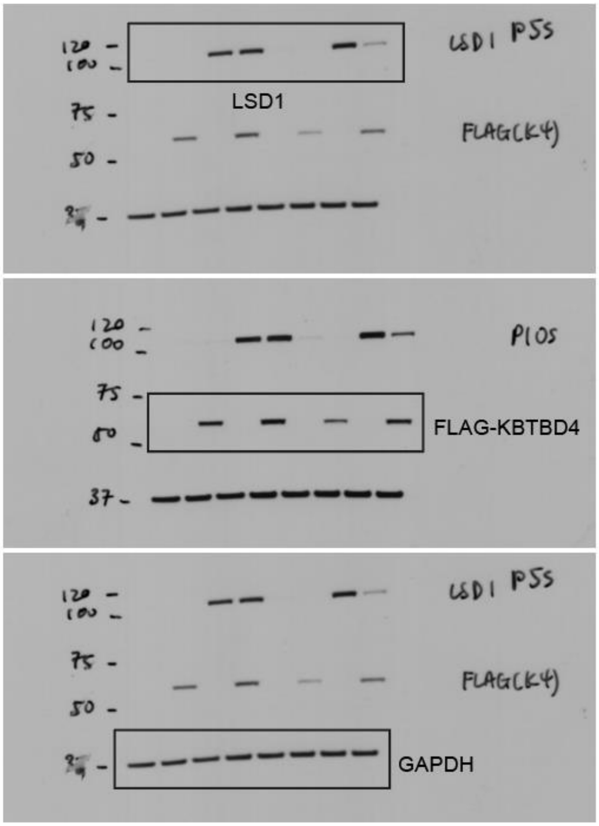

ED Fig 2f - same blots different exposure

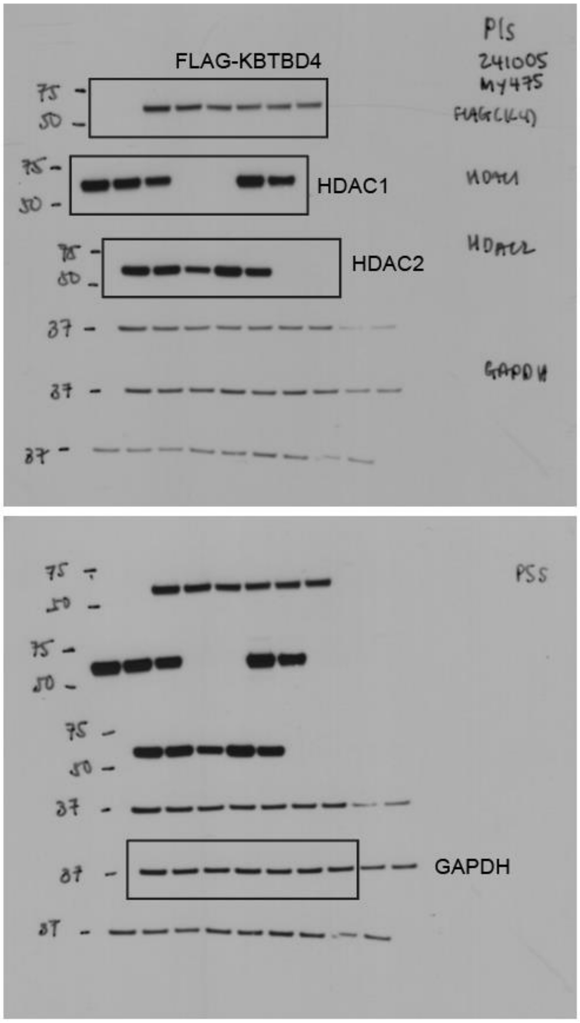

ED Fig 2g - same blots different exposure

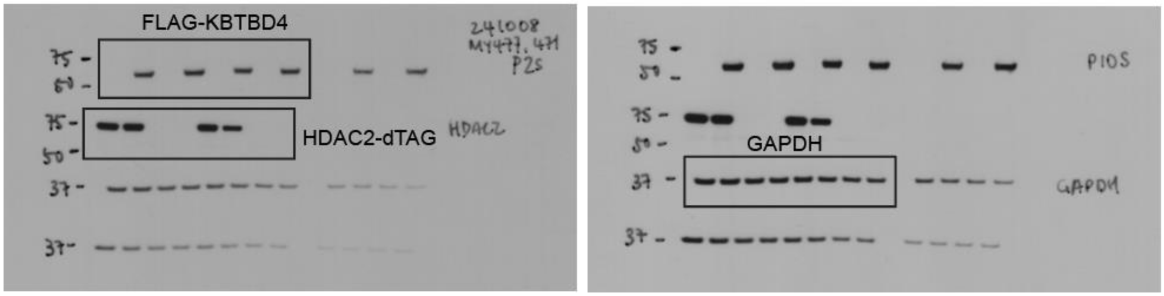

Supplementary Figure 6 | Uncropped western blot and gel images in ED Fig. 3

ED Fig 3b

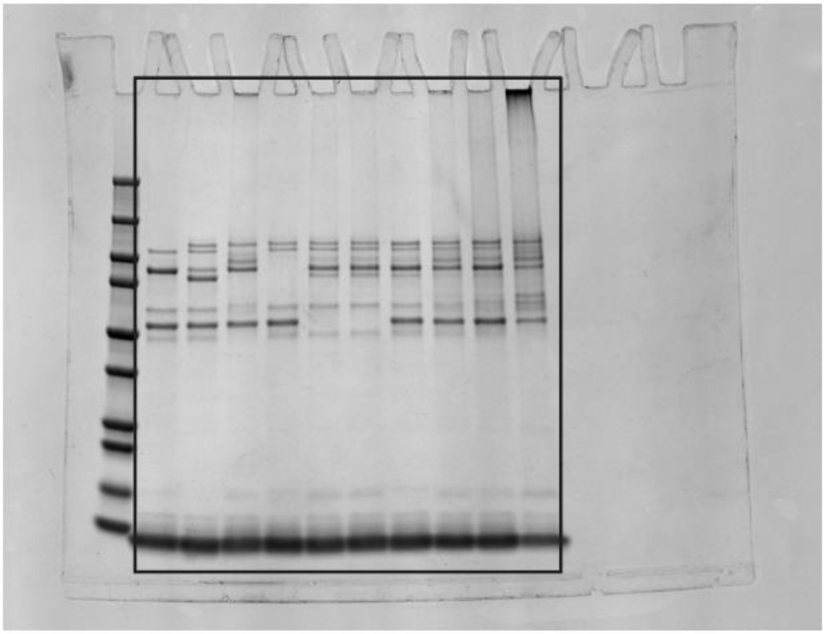

ED Fig 3c

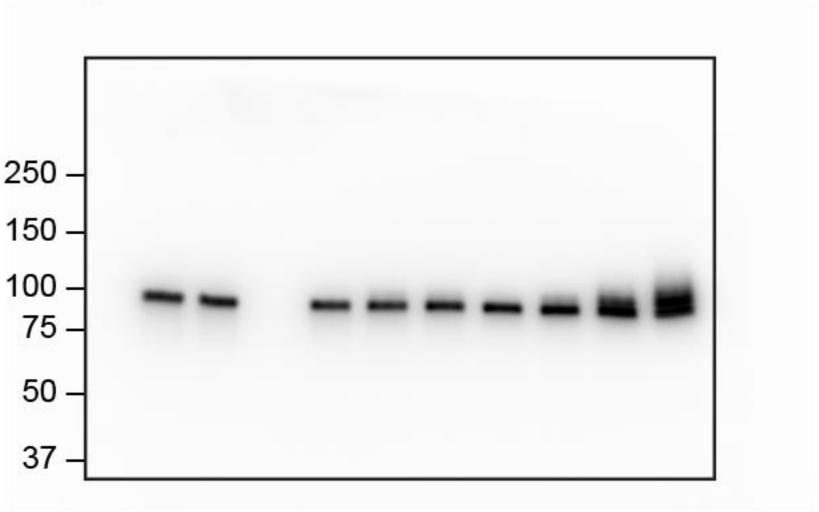

Supplementary Figure 7 | Uncropped western blot images in Extended Data Figure 10

ED Fig 10d

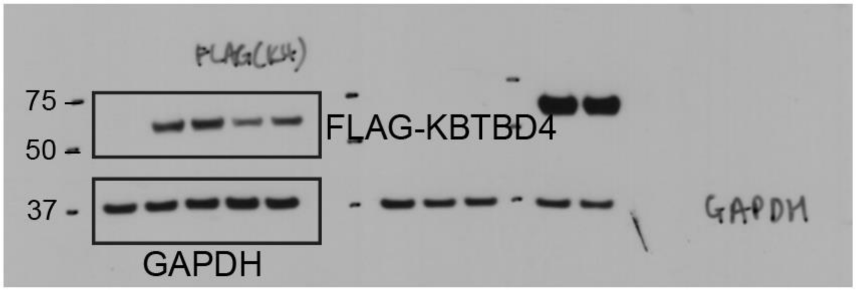

ED Fig 10g

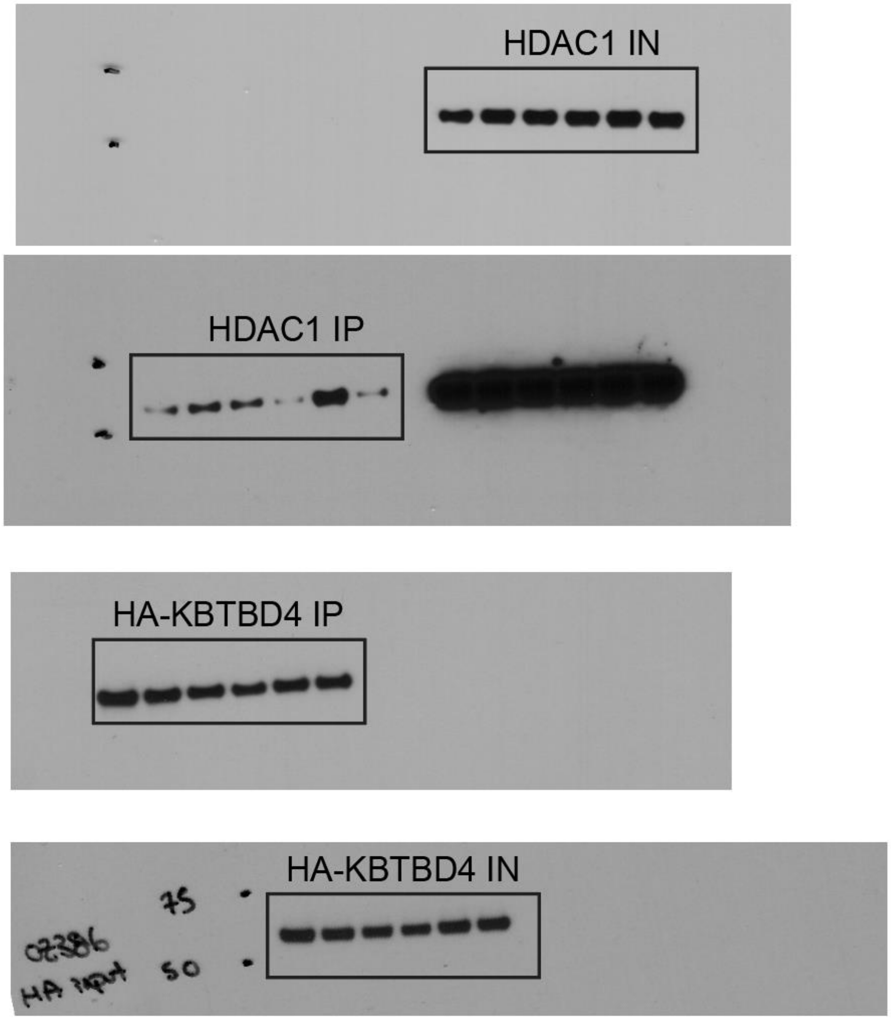

Supplementary Table 1 | sgRNA sequences for eVLP production
All oligonucleotides were obtained from Genewiz.

Vector	Gene	sgRNA	Protospacer	Predicted amino acid edits
pU6-sgRNA (for eVLPs)	KBTBD4	sgY120	CATGGTAGATATAATCAACC	Tyr120His
	KBTBD4	sgC297	GCTTGCAGGCCGCAGTGATC	Cys297Arg
	KBTBD4-PR	sgM316	TCCACATGCGCCGTGGCCGT	Trp315Arg;Met314 Thr
	HEK3	sgHEK3	GGCCCAGACTGAGCACGTGA	-

Supplementary Table 2 | Sequencing primers for base editing validation
All primers were obtained from Sigma-Aldrich.

Name	Primer sequence	Target
KBTBD4_Y120_SF1	ACACTCTTTCCCTACACGACGCTCTTCCGATCTNNNNNCCGAGAGCATTCCTCAAAGA	KBTBD4 sgY120
KBTBD4_Y120_SR1	TGGAGTTCAGACGTGTGCTCTTCCGATCTTCAGCGAGTCTGTTTTCCAG	
KBTBD4_C297_SF1	ACACTCTTTCCCTACACGACGCTCTTCCGATCTNNNNNGCACTTCCACATGCGCCGTG	KBTBD4 sgC297
KBTBD4_C297_SR1	TGGAGTTCAGACGTGTGCTCTTCCGATCTACCCACTCGTTGGCTGTGTCC	
KBTBD4_M316_SF1	ACACTCTTTCCCTACACGACGCTCTTCCGATCTNNNNNCACACCACTCCCAGTCAAC	KBTBD4-PR sgM316
KBTBD4_M316_SR1	TGGAGTTCAGACGTGTGCTCTTCCGATCTAGTTTGTGCCACCAGATCAC	
HEK3_SF1	ACACTCTTTCCCTACACGACGCTCTTCCGATCTNNNNNTGCATTTGTAGGCTTGATGCT	sgHEK3
HEK3_SR1	TGGAGTTCAGACGTGTGCTCTTCCGATCTGGAGCTGCACATACTAGCCC	

Primer binding sequence
Overhang for amplification with P5 site primer
Overhang for amplification with P7 site primer

Supplementary Table 3 | Sequencing primers for knockout experiments
All primers were obtained from Sigma-Aldrich.

Name	Primer sequence	Target
HDAC1_sg2_SF3	ACACTCTTTCCCTACACGACGCTCTTCCGATCTNNNNC CAGGTAGCACAAGGATGGG	HDAC1 I305
HDAC1_sg2_SR3	TGGAGTTCAGACGTGTGCTCTTCCGATCTATATGACCA ACGGGGAAGGG	
HDAC2_sg2_SF1	ACACTCTTTCCCTACACGACGCTCTTCCGATCTNNNNA CTGATCTCCTAGGTTCATCTCA	HDAC2 M195
HDAC2_sg1_SR1	TGGAGTTCAGACGTGTGCTCTTCCGATCTACCAAAACT TATGGATTTGTTTTCAGG	
KBTBD4_sg4_SF1	ACACTCTTTCCCTACACGACGCTCTTCCGATCTNNNNG TAAACTAACACCAGGAAGCTTGA	KBTBD4 L190
KBTBD4_sg4_SR1	TGGAGTTCAGACGTGTGCTCTTCCGATCTCCAAGCACT GTGCCAAGA	
LSD1_sgN660_SF1	ACACTCTTTCCCTACACGACGCTCTTCCGATCTNNNNT GCCACCTCTCCCTGAGTGGA	LSD1 N660
LSD1_sgN660_SR1	TGGAGTTCAGACGTGTGCTCTTCCGATCTTGCAAGGC CACCTCCTCACC	

Primer binding sequence
Overhang for amplification with P5 site primer
Overhang for amplification with P7 site primer

Supplementary Table 4 | sgRNA sequences for knockout experiments
All primers were obtained from Sigma-Aldrich.

Name	Protospacer	Target Residue
HDAC1_KO_sgRNA	GCACCGGGCAACGTTACGAA	I305
HDAC2_KO_sgRNA	TACAACAGATCGTGTAATGA	M195
KBTBD4_KO_sgRNA	GATATCTGTGAGTAAGCGGT	L190
LSD1_KO_sgRNA	TAGGGCAAGCTACCTTGTTA	N660
sgControl	CCCGGCGCCATTCTATCCGC	Luciferase (control)

Supplementary Table 5 | Primers used for DMS library cloning
All oligonucleotides were obtained from Sigma-Aldrich.

Name	Sequence (5’ to 3’)
KBTBD4_lsPCR2_Fw	aacagtttgtgccaccagatcactgcggcctgcaagcatggtggagactt
KBTBD4_lsPCR2_Rv	tcccgaggcaaaggagcacaccactcccagtcaacggtggcattgttgc
KBTBD4_lsPCR1_BC1_Fw	CGGGTTCCGTtgcaagca
KBTBD4_lsPCR1_BC1_Rv	TTCTATTCTAAGCgtcaacggtgg
KBTBD4_lsPCR1_BC2_Fw	GTTTATCGGGCtgcaagcatg
KBTBD4_lsPCR1_BC2_Rv	GGTACAGTAAGTgtcaacggtgg
KBTBD4_lsPCR1_BC3_Fw	ACCGATGTTGACtgcaagca
KBTBD4_lsPCR1_BC3_Rv	GCTATTACGAGgtcaacggtgg
KBTBD4_lsPCR1_BC4_Fw	GAGGTCTTTCATGCtgcaagc
KBTBD4_lsPCR1_BC4_Rv	TATGTTGTGgtcaacggtggc
KBTBD4_lsPCR1_BC5_Fw	TATCCCGTGAAGCTtgcaag
KBTBD4_lsPCR1_BC5_Rv	TTAACCGAAgtcaacggtgg
KBTBD4_lsPCR1_BC6_Fw	TAGTAGTTCAGACGCtgcaagc
KBTBD4_lsPCR1_BC6_Rv	GGGTACATgtcaacggtggc

Supplementary Table 6 | Primers used for DMS plasmid construction
All primers were obtained from Sigma-Aldrich.

Name	Sequence (5’ to 3’)
PacI_Fw	acaacccctcactcggcttaattaa
BamHI_Rv	cacttccacatGGATCcaccacatacaagtctccaccatgcttgc
BamHI_Fw	ggtgGATCCatgtggaagtgcaacaatgccac
KpnI_Rv	gccttgtaagtcattggtcttaaaggtac

Supplementary Table 7 | Primers used for DMS sequencing
All oligonucleotides were obtained from Sigma-Aldrich.

Name	Sequence (5' to 3')
pSMAL_KBTBD4_P5_S1_w_intron	AATGATACGGCGACCACCGAGATCTACACTCTTTCCCTACACGACGCTCTTCCGATCTagtcaacggtggcattgttgc
pSMAL_KBTBD4_P5_S2_w_intron	AATGATACGGCGACCACCGAGATCTACACTCTTTCCCTACACGACGCTCTTCCGATCTCagtcaacggtggcattgttgc
pSMAL_KBTBD4_P5_S3_w_intron	AATGATACGGCGACCACCGAGATCTACACTCTTTCCCTACACGACGCTCTTCCGATCTGCagtcaacggtggcattgttgc
pSMAL_KBTBD4_P5_S4_w_intron	AATGATACGGCGACCACCGAGATCTACACTCTTTCCCTACACGACGCTCTTCCGATCTAGCagtcaacggtggcattgttgc
pSMAL_KBTBD4_P5_S5_w_intron	AATGATACGGCGACCACCGAGATCTACACTCTTTCCCTACACGACGCTCTTCCGATCTCAACagtcaacggtggcattgttgc
pSMAL_KBTBD4_P5_S6_w_intron	AATGATACGGCGACCACCGAGATCTACACTCTTTCCCTACACGACGCTCTTCCGATCTTGCACCagtcaacggtggcattgttgc
pSMAL_KBTBD4_P5_S7_w_intron	AATGATACGGCGACCACCGAGATCTACACTCTTTCCCTACACGACGCTCTTCCGATCTACGCAACagtcaacggtggcattgttgc
pSMAL_KBTBD4_P5_S8_w_intron	AATGATACGGCGACCACCGAGATCTACACTCTTTCCCTACACGACGCTCTTCCGATCTGAAGACCCagtcaacggtggcattgttgc
pSMAL_KBTBD4_P7_A1_w_intron	CAAGCAGAAGACGGCATACGAGATCGGTTCAAGTGACTGGAGTTCAGACGTGTGCTCTTCCGATCTtgcagagtcactcaggacaagcttg
pSMAL_KBTBD4_P7_A2_w_intron	CAAGCAGAAGACGGCATACGAGATGCTGGATTGTGACTGGAGTTCAGACGTGTGCTCTTCCGATCTtgcagagtcactcaggacaagcttg
pSMAL_KBTBD4_P7_A3_w_intron	CAAGCAGAAGACGGCATACGAGATTAACTCGGGTGACTGGAGTTCAGACGTGTGCTCTTCCGATCTtgcagagtcactcaggacaagcttg
pSMAL_KBTBD4_P7_A4_w_intron	CAAGCAGAAGACGGCATACGAGATTAACAGTTGTGACTGGAGTTCAGACGTGTGCTCTTCCGATCTtgcagagtcactcaggacaagcttg
pSMAL_KBTBD4_P7_A5_w_intron	CAAGCAGAAGACGGCATACGAGATATACTCAAGTGACTGGAGTTCAGACGTGTGCTCTTCCGATCTtgcagagtcactcaggacaagcttg
pSMAL_KBTBD4_P7_A6_w_intron	CAAGCAGAAGACGGCATACGAGATGCTGAGAAGTGACTGGAGTTCAGACGTGTGCTCTTCCGATCTtgcagagtcactcaggacaagcttg
pSMAL_KBTBD4_P7_A7_w_intron	CAAGCAGAAGACGGCATACGAGATATTGGAGGGTGACTGGAGTTCAGACGTGTGCTCTTCCGATCTtgcagagtcactcaggacaagcttg
pSMAL_KBTBD4_P7_A8_w_intron	CAAGCAGAAGACGGCATACGAGATTAGTCTAAGTGACTGGAGTTCAGACGTGTGCTCTTCCGATCTtgcagagtcactcaggacaagcttg
pSMAL_KBTBD4_P7_A9_w_intron	CAAGCAGAAGACGGCATACGAGATCGGTGACCGTGACTGGAGTTCAGACGTGTGCTCTTCCGATCTtgcagagtcactcaggacaagcttg
pSMAL_KBTBD4_P7_A10_w_intron	CAAGCAGAAGACGGCATACGAGATTACAGAGGGTGACTGGAGTTCAGACGTGTGCTCTTCCGATCTtgcagagtcactcaggacaagcttg
pSMAL_KBTBD4_P7_A11_w_intron	CAAGCAGAAGACGGCATACGAGATATTGTCAAGTGACTGGAGTTCAGACGTGTGCTCTTCCGATCTtgcagagtcactcaggacaagcttg
pSMAL_KBTBD4_P7_A12_w_intron	CAAGCAGAAGACGGCATACGAGATTATGTCTTGTGACTGGAGTTCAGACGTGTGCTCTTCCGATCTtgcagagtcactcaggacaagcttg

pSMAL_KBTBD4_P7_B1_w_intron	CAAGCAGAAGACGGCATACGAGATATTGGATTGTGACTGGAGTTCAGACGTGTGCTCTTCCGATCTtgcagagtcactcaggacaagcttg
pSMAL_KBTBD4_P7_B2_w_intron	CAAGCAGAAGACGGCATACGAGATATACTCGGGTGACTGGAGTTCAGACGTGTGCTCTTCCGATCTtgcagagtcactcaggacaagcttg
pSMAL_KBTBD4_P7_B3_w_intron	CAAGCAGAAGACGGCATACGAGATTATGAGAAGTGACTGGAGTTCAGACGTGTGCTCTTCCGATCTtgcagagtcactcaggacaagcttg
pSMAL_KBTBD4_P7_B4_w_intron	CAAGCAGAAGACGGCATACGAGATGCACAGTTGTGACTGGAGTTCAGACGTGTGCTCTTCCGATCTtgcagagtcactcaggacaagcttg
pSMAL_KBTBD4_P7_B5_w_intron	CAAGCAGAAGACGGCATACGAGATCGTGGATTGTGACTGGAGTTCAGACGTGTGCTCTTCCGATCTtgcagagtcactcaggacaagcttg
pSMAL_KBTBD4_P7_B6_w_intron	CAAGCAGAAGACGGCATACGAGATTAGTAGAAGTGACTGGAGTTCAGACGTGTGCTCTTCCGATCTtgcagagtcactcaggacaagcttg

Supplementary Methods

Synthetic Procedures

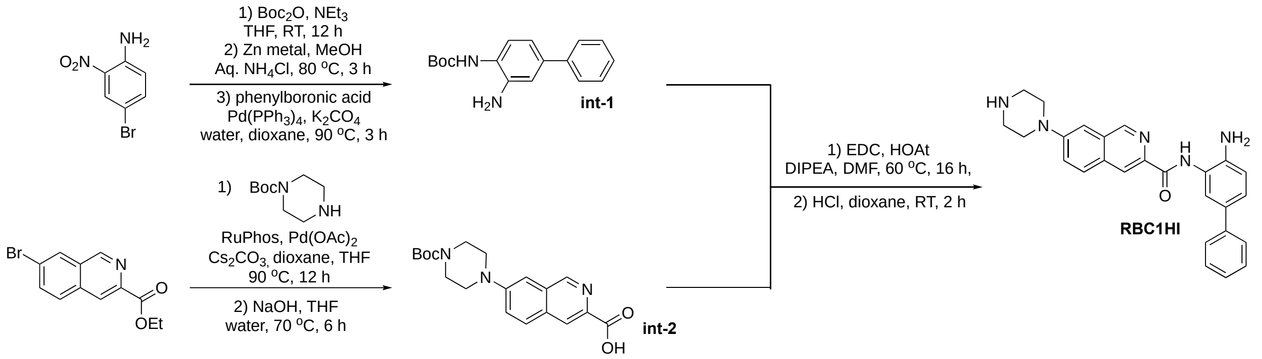

<u>Synthesis of intermediate 1 (**int-1**):</u>

4-Bromo-2-nitroaniline (5.00 g, 10.9 mmol) was dissolved in THF (100 mL) along with triethylamine (3.03 mL, 21.7 mmol). Subsequently, Boc_2O (4.74 g, 21.7 mmol) was added portion wise. The resulting solution was stirred at room temperature for 12 h and was then concentration in vacuo. The concentrated solution was redissolved in ethyl acetate (EtOAc) and transferred to a separatory funnel. The organic layers were washed with water, then dried over magnesium sulfate, and then concentrated to yield a crude product that was carried forward without additional purification.

tert-butyl (4-bromo-2-nitrophenyl)carbamate (1.00 g, 3.15 mmol) was dissolved in MeOH (50 mL) and saturated aqueous ammonium chloride (15 mL). Zinc powder (1.02 g, 15.8 mmol) was then added, and the reaction mixture was heated to 80 °C for 3 h. The slurry was filtered through celite, the resultant filtrate was concentrated. The resulting crude product was redissolved in EtOAc, washed with water, dried over magnesium sulfate, and concentrated. Purification by column chromatography on silica gel yielded *tert*-butyl (2-amino-4-bromophenyl)carbamate (81%, 0.724 g) as a yellow solid.

A mixture of *tert*-butyl (2-amino-4-bromophenyl)carbamate (1.00 g, 3.48 mmol), phenylboronic acid (0.506 g, 4.18 mmol), and K_2CO_3 (2M in water, 10 mL) in dioxane (20 mL) was sparged with N_2, followed by the addition of $Pd(PPh_3)_4$ (0.121 g, 0.105 mmol). The reaction mixture was stirred at 90 °C for 6 h. After cooling, the crude mixture was extracted with EtOAc, washed with water and brine, and then concentrated in vacuo. Purification by column chromatography on silica gel yielded *tert*-butyl (3-amino-[1,1'-biphenyl]-4-yl)carbamate (**int-1**) (95%, 0.940 g) as a brown solid.

<u>Synthesis of intermediate 2 (**int-2**):</u>

The pre-complexation of $Pd(OAc)_2$ (0.240 g, 0.300 mmol) and RuPhos (0.600 g, 0.400 mmol) proceeded by stirring in 5 mL of degassed THF at 40 °C for 1 h. A mixture comprising ethyl 7-bromoisoquinoline-3-carboxylate (1.00 g, 3.57 mmol), *tert*-butyl piperazine-1-carboxylate (0.798 g, 4.29 mmol and Cs_2CO_3 (1.74 g, 5.36 mmol) in degassed dioxane (30 mL) was then added to the activated catalyst and stirred at 90 °C overnight. After cooling, the mixture was filtered, concentrated in vacuo, and then purified by column chromatography to yield ethyl 7-(4-(*tert*-butoxycarbonyl)piperazin-1-yl)isoquinoline-3-carboxylate (94%, 0.646 g) as a yellow solid.

A solution of ethyl 7-(4-(*tert*-butoxycarbonyl)piperazin-1-yl)isoquinoline-3-carboxylate (0.500 g, 1.30 mmol) in a mixture of MeOH (5 mL) and THF (5 mL) was combined with aqueous 1M NaOH (5 mL) and stirred at 60 °C for 3 h. The resulting mixture was concentrated and the residue was taken up in water (20 ml), and the pH was acidified using 2M HCl. A yellow solid

precipitated, which was separated by filtration, washed with water (50 ml), and then dried, yielding 7-(4-(*tert*-butoxycarbonyl)piperazin-1-yl)isoquinoline-3-carboxylic acid (**int-2**) (80%, 0.370 g) as a yellow solid.

Synthesis of RBC1HI:

A mixture comprising 7-(4-(*tert*-butoxycarbonyl)piperazin-1-yl)isoquinoline-3-carboxylic acid (0.100 g, 0.280 mmol), *tert*-butyl (3-amino-[1,1'-biphenyl]-4-yl)carbamate (0.110 g, 0.386 mmol), EDC (0.0738 g, 0.386 mmol), HOAt (0.0524 g, 0.386 mmol), and DIPEA (0.122 mL, 0.702 mmol) in DMF (2 mL) was stirred at room temperature for 16 h. The solution was then concentrated in vacuo and the resultant residue was purified by silica gel column chromatography, to yield *tert*-butyl 4-(3-((4-((tert-butoxycarbonyl)amino)-[1,1'-biphenyl]-3-yl)carbamoyl)isoquinolin-7-yl)piperazine-1-carboxylate (80%, 0.139 g) as a yellow solid.

tert-butyl 4-(3-((4-((*tert*-butoxycarbonyl)amino)-[1,1'-biphenyl]-3-yl)carbamoyl)isoquinolin-7-yl)piperazine-1-carboxylate was treated with 4N HCl in dioxane (5 mL) and stirred at room temperature for 2 h, and then concentrated in vacuo. The resultant solid was washed with diethyl ether (20 ml) and subjected to C18 column chromatography to provide RBC1HI•HCl salt (64%, 0.070 g) as a yellow solid. ^{1}H NMR (400 MHz, METHANOL-D4): δ 9.45 (s, 1H), 8.96 (s, 1H), 8.24 (d, J = 9.3 Hz, 1H), 8.01 (d, J = 8.5 Hz, 1H), 7.89 (d, J = 2.1 Hz, 1H), 7.78 (m, 2H), 7.71 (m, 2H), 7.63 (d, J = 8.3 Hz, 1H), 7.50 (m, 2H), 7.43 (m, 1H), 3.80 (m, 4H), 3.48 (m, 4H). ^{13}C NMR (101 MHz, METHANOL-D4) δ 160.74, 152.03, 146.78, 142.70, 138.40, 131.38, 131.18, 130.96, 130.52, 130.04, 128.87, 128.17, 127.46, 126.73, 126.20, 125.09, 124.86, 124.76, 124.36, 110.11, 44.50, 43.03. HRMS-ESI [M+H]: expected mass: 424.2137 and observed: 424.2133

NMR Spectra of RBC1HI•HCl

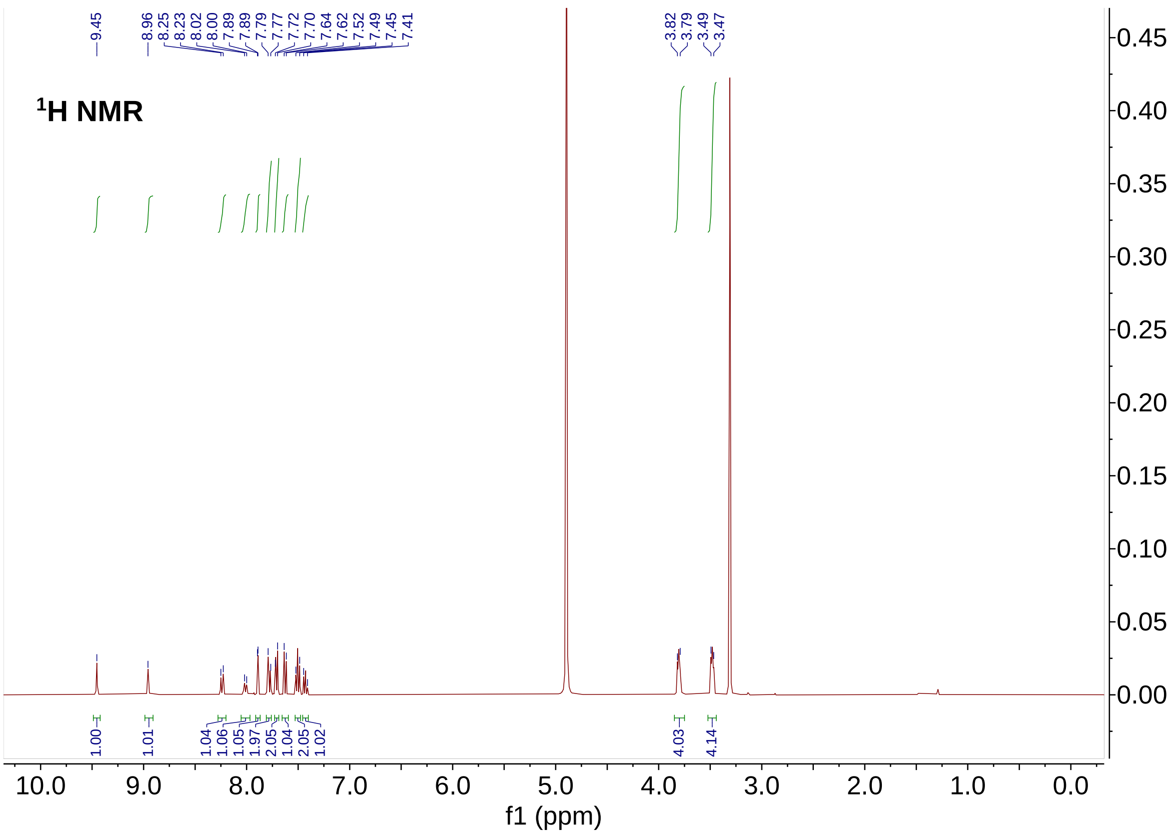

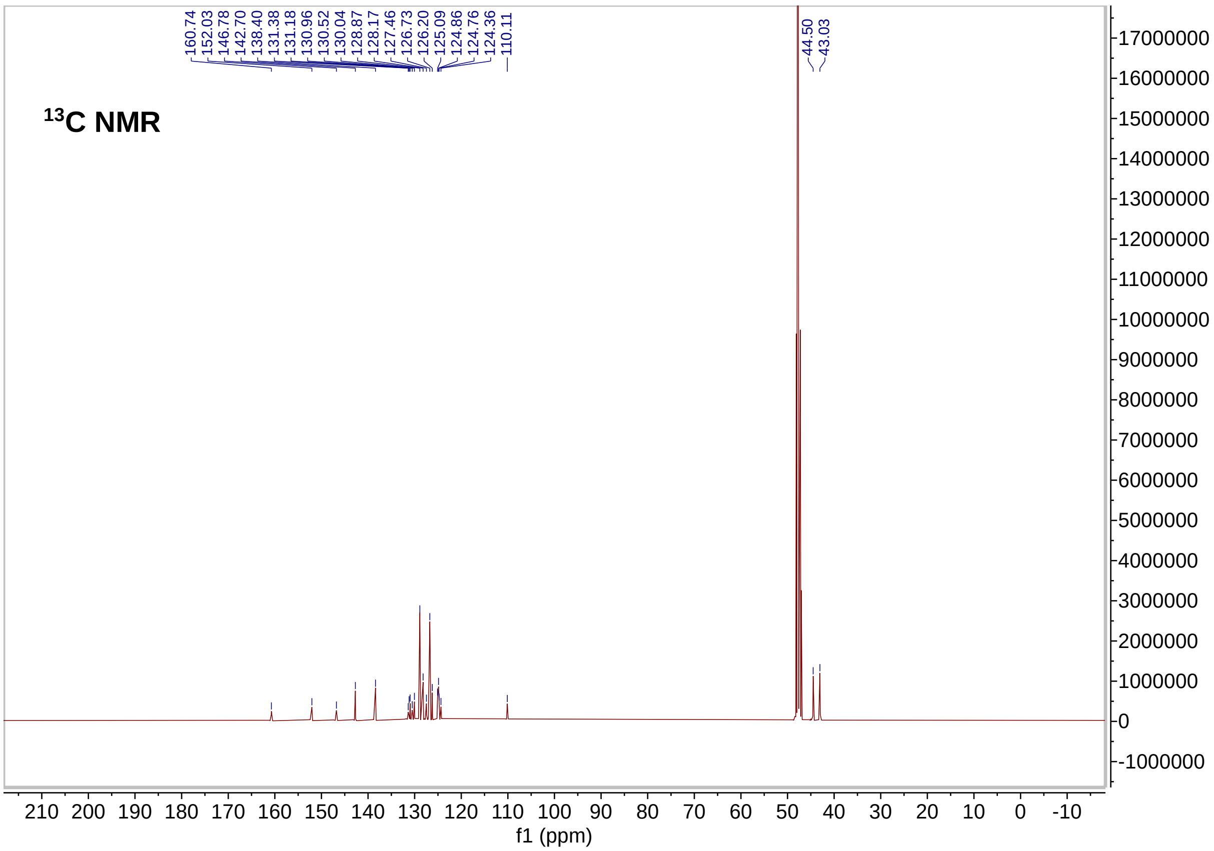

Supplement: Xie et al Supplementary Information [file NIHMS2059354-supplement-Xie_et_al_Supplementary_Information.pdf]
