# Supplementary material for: Converging mechanism of UM171 and KBTBD4 neomorphic cancer mutations
Source: Nature. 2025 Feb 12;639(8053):241–9. doi: 10.1038/s41586-024-08533-3 (PMC11882451; doi:10.1038/s41586-024-08533-3)
Supplement: Supplementary file 1 [file 41586_2024_8533_MOESM2_ESM.pdf]

# nature portfolio

Corresponding author(s): Ning Zheng, Brian Liau

Last updated by author(s): Nov 24, 2024

# Reporting Summary

Nature Portfolio wishes to improve the reproducibility of the work that we publish. This form provides structure for consistency and transparency in reporting. For further information on Nature Portfolio policies, see our Editorial Policies and the Editorial Policy Checklist.

## Statistics

For all statistical analyses, confirm that the following items are present in the figure legend, table legend, main text, or Methods section.

| n/a | Confirmed | |
|---|---|---|
| ☐ | ☒ | The exact sample size (*n*) for each experimental group/condition, given as a discrete number and unit of measurement |
| ☐ | ☒ | A statement on whether measurements were taken from distinct samples or whether the same sample was measured repeatedly |
| ☐ | ☒ | The statistical test(s) used AND whether they are one- or two-sided<br>*Only common tests should be described solely by name; describe more complex techniques in the Methods section.* |
| ☒ | ☐ | A description of all covariates tested |
| ☒ | ☐ | A description of any assumptions or corrections, such as tests of normality and adjustment for multiple comparisons |
| ☐ | ☒ | A full description of the statistical parameters including central tendency (e.g. means) or other basic estimates (e.g. regression coefficient) AND variation (e.g. standard deviation) or associated estimates of uncertainty (e.g. confidence intervals) |
| ☐ | ☒ | For null hypothesis testing, the test statistic (e.g. *F*, *t*, *r*) with confidence intervals, effect sizes, degrees of freedom and *P* value noted<br>*Give P values as exact values whenever suitable.* |
| ☒ | ☐ | For Bayesian analysis, information on the choice of priors and Markov chain Monte Carlo settings |
| ☒ | ☐ | For hierarchical and complex designs, identification of the appropriate level for tests and full reporting of outcomes |
| ☐ | ☒ | Estimates of effect sizes (e.g. Cohen's *d*, Pearson's *r*), indicating how they were calculated |

*Our web collection on statistics for biologists contains articles on many of the points above.*

## Software and code

Policy information about availability of computer code

| | |
|---|---|
| Data collection | Flow cytometry data were collected using a NovoCyte 3000RYB flow cytometer and the NovoExpress software (v1.6.1). TR-FRET assay data were collected using a Tecan SPARK plate reader with SPARKCONTROL software version V2.1 (Tecan Group Ltd.). Cell viability assay data were collected using a PHERAstar FSX microplate reader.<br><br>Deep mutational scanning was sequenced on an Illumina Miseq instrument.<br>Cryo-EM: Glacios Transmission Electron Microscope (Thermo Fisher) with K3 direct electron detector, operated on SerialEM software (v4.1.8), and FEI Titan Krios Transmission Electron Microscope (Thermo Fisher) with K3 direct electron detector, operated on SerialEM software. |
| Data analysis | Proteomics data were analyzed and visualized using R environment (version 4.3.2) with the limma R package (version 3.54.2) and R package ggplot2 (version 3.5.0), STRINGdb (version 12), and Cytoscape (version 3.5.10).<br><br>Deep mutational scanning data were analyzed using Python (v3.9.12) with the following packages: Biopython (v.1.78), Pandas (v.1.5.1), NumPy (v.1.23.4), matplotlib (v3.7.1), Logomaker (v0.8). Flow cytometry data were analyzed using NovoExpress (v1.6.1). Other data were analyzed using Microsoft Excel (v16.80) and GraphPad Prism (v9.4.0 and v10.1.1).<br>Data were visualized using NovoExpress (v1.6.1), GraphPad Prism, and Adobe Illustrator 2022 (v26.0.3). Structural analysis and visualization was performed using PyMOL (v2.5.4).<br><br>CryoEM data was analyzed with CryoSPARC(4.4.1); the model building is based on a primary model predicted with AlphaFold-Multimer in Google ColabFold2, then further modified in ChimeraX-1.7 (rc2023.12.12), PHENIX (1.20.1-4487-000) and Coot (0.9.8.91). |

For manuscripts utilizing custom algorithms or software that are central to the research but not yet described in published literature, software must be made available to editors and reviewers. We strongly encourage code deposition in a community repository (e.g. GitHub). See the Nature Portfolio guidelines for submitting code & software for further information.

## Data

Policy information about availability of data

All manuscripts must include a data availability statement. This statement should provide the following information, where applicable:

- Accession codes, unique identifiers, or web links for publicly available datasets
- A description of any restrictions on data availability
- For clinical datasets or third party data, please ensure that the statement adheres to our policy

The coordinates and density map of the KBTBD4-PR-LHC-InsP6, KBTBD4-TTYML-LHC-InsP6, and KBTBD4-PR-HDAC2-CoREST complexes are deposited in the Protein Data Bank (PDB) with the accession numbers 8VRT, 8VPQ, and 9DTQ, and in the Electron Microscopy Data Bank (EMDB) with the accession numbers EMD-43487, EMD-43413, and EMD-47156, respectively. DepMap (24Q4 release) was downloaded from https://depmap.org/portal/. The following publicly available datasets were used: PDB accession codes 4LXZ. MS-based proteomics raw data files, DMS data, oligonucleotide sequences, as well as additional data generated by this study are provided as Supplementary Information and Source data.

## Research involving human participants, their data, or biological material

Policy information about studies with human participants or human data. See also policy information about sex, gender (identity/presentation), and sexual orientation and race, ethnicity and racism.

| | |
|---|---|
| Reporting on sex and gender | N/A |
| Reporting on race, ethnicity, or other socially relevant groupings | N/A |
| Population characteristics | N/A |
| Recruitment | N/A |
| Ethics oversight | N/A |

Note that full information on the approval of the study protocol must also be provided in the manuscript.

# Field-specific reporting

Please select the one below that is the best fit for your research. If you are not sure, read the appropriate sections before making your selection.

☒ Life sciences ☐ Behavioural & social sciences ☐ Ecological, evolutionary & environmental sciences

For a reference copy of the document with all sections, see nature.com/documents/nr-reporting-summary-flat.pdf

# Life sciences study design

All studies must disclose on these points even when the disclosure is negative.

| | |
|---|---|
| Sample size | No statistical methods were used to determine sample size. For all cellular assays we used a minimum sample size of n = 3 replicates and for all biochemical assays we used a minimum sample size of n = 2 replicates and further confirmed reproducibility by replicating each experiment in two independent trials unless otherwise noted. This yields reproducible results based on our experience and is standard for cellular and biochemical assays (Vinyard et al., 2019). For immunoblotting experiments, we conducted these in singlicate for practical purposes and further confirmed reproducibility by replicating each experiment twice unless otherwise noted. This is standard practice for assays involving gels or blotting (Vinyard et al., 2019). This yielded reproducible results. For deep mutational scanning, we used a sample size of n = 3 replicates and additionally ensured that the number of cells and sequencing depth maintained >150X coverage of the mutational library. |
| Data exclusions | No data was excluded. |
| Replication | Where indicated in the paper, experiments were performed in replicate (duplicate or triplicate). Replicate type is specified in the text. All attempts at replication were successful. |
| Randomization | No randomization was performed as this was not applicable to the experiments performed in this study. None of the experiments performed in this study involved allocating discrete samples or organisms to experimental groups. For example, for cell culture experiments, aliquots of cells from a common parent culture were typically seeded into separate flasks/wells for transfection with different plasmids or transduction with different viruses. |
| Blinding | Investigators were not blinded for any of the experiments performed in this study as knowledge of the sample does not affect machine-based measurement of these data. This was done for practical purposes, and is standard practice for studies employing biochemistry, cell culture, and genomics. |

# Reporting for specific materials, systems and methods

We require information from authors about some types of materials, experimental systems and methods used in many studies. Here, indicate whether each material, system or method listed is relevant to your study. If you are not sure if a list item applies to your research, read the appropriate section before selecting a response.

## Materials & experimental systems

| n/a | Involved in the study | |
|---|---|---|
| ☐ | ☒ | Antibodies |
| ☐ | ☒ | Eukaryotic cell lines |
| ☒ | ☐ | Palaeontology and archaeology |
| ☐ | ☒ | Animals and other organisms |
| ☒ | ☐ | Clinical data |
| ☒ | ☐ | Dual use research of concern |
| ☒ | ☐ | Plants |

## Methods

| n/a | Involved in the study | |
|---|---|---|
| ☒ | ☐ | ChIP-seq |
| ☐ | ☒ | Flow cytometry |
| ☒ | ☐ | MRI-based neuroimaging |

## Antibodies

Antibodies used

LSD1 (Bethyl Laboratories, A300-215A, Lot no. 2)
RCOR1 (Cell Signaling Technology, #14567, Lot no. 1)
GAPDH (Santa Cruz Biotechnology, sc-477724, Lot no. G2920; RRID: AB_627678)
HA (Cell Signaling Technology, #3724, Lot no. 10)
FLAG (Sigma-Aldrich, F1804, Lot no. #SLCN3722)
KBTBD4 (Novus Biologicals, NBP1-88587, Lot no. A116815 )
HDAC1 (Cell Signaling Technology, #34589, Lot no. 4)
HDAC2 (Cell Signaling Technology, #57156, Lot no. 1)
Anti-Rabbit IgG HRP conjugate (Promega, Cat#W4011, RRID:AB_430833)
Anti-Mouse IgG HRP conjugate (Promega, Cat#W4021, RRID:AB_430834)
Pierce™ Anti-HA Magnetic Beads (Thermo Scientific, #88837)

Validation

All antibodies used were commercial and validated for the appropriate application.
LSD1 (Bethyl Laboratories, A300-215A, Lot no. 2) validated by manufacturer and citations at https://www.fortislife.com/cms/files/A300-215A-2.pdf
RCOR1 (Cell Signaling Technology, #14567, Lot no. 1) validated by manufacturer and citations at https://www.cellsignal.com/products/14567/datasheet?images=1&protocol=0
GAPDH (Santa Cruz Biotechnology, sc-477724, Lot no. G2920; RRID: AB_627678) validated by manufacturer and citations at https://datasheets.scbt.com/sc-47724.pdf
HA (Cell Signaling Technology, #3724, Lot no. 10) validated by manufacturer and citations at https://awsqa-www.cellsignal.com/datasheet.jsp?productId=3724&images=1
FLAG (Sigma-Aldrich, F1804, Lot no. #SLCN3722) validated by manufacturer and citations at https://www.sigmaaldrich.com/certificates/sapfs/PROD/sap/certificate_pdfs/COFA/Q14/F1804-BULKSLCN3722.pdf
KBTBD4 (Novus Biologicals, NBP1-88587, Lot no. A116815) validated by manufacturer and citations at https://www.novusbio.com/PDFs/NBP1-88587.pdf
HDAC1 (Cell Signaling Technology, #34589, Lot no. 4) validated by manufacturer and citations at https://www.cellsignal.com/products/34589/datasheet?images=1&protocol=0
HDAC2 (Cell Signaling Technology, #57156, Lot no. 1) validated by manufacturer and citations at https://www.cellsignal.com/products/57156/datasheet?images=1&protocol=0
Anti-Rabbit IgG HRP conjugate (Promega, Cat#W4011, RRID:AB_430833) validated by manufacturer and citations at https://www.promega.com/en/products/protein-detection/primary-and-secondary-antibodies/anti-rabbit-igg-h-and-l-hrp-conjugate/?catNum=W4011#resources
Anti-Mouse IgG HRP conjugate (Promega, Cat#W4021, RRID:AB_430834) validated by manufacturer and citations at https://www.promega.com/products/protein-detection/primary-and-secondary-antibodies/anti_mouse-igg-h-and-l-hrp-conjugate/?catNum=W4021
Pierce™ Anti-HA Magnetic Beads (Thermo Scientific, #88837) validated by the manufacturer and citations at https://www.thermofisher.com/order/catalog/product/88837

## Eukaryotic cell lines

Policy information about cell lines and Sex and Gender in Research

Cell line source(s)

HEK293T (Thermo Fisher) was a gift from Bradley E. Bernstein;  K562 was obtained from ATCC (CCL-243); HEK293F was obtained from Thermo Fisher (CVCL_6642); Gesicle Producer 293T cells were a gift from David R. Liu (Takara, 632617); CHLA-01-MED was obtained from ATCC (CRL-3021); RCMB51, RCMB52, and RCMB28 were originated and shared by Robert J. Wechsler-Reya, Ph.D., Columbia University (previously Stanford); ICB1299 and ICB1572 were originated and shared by Xiao-Nan Li, M.D., Ph.D., Northwestern University Feinberg School of Medicine (previously Baylor University); MED411FH, MED411FH-TC (established for tissue culture), MED2312FH, MED211FH, and MED2312FH were obtained from Brain Tumor Research Laboratory, Seattle Children's Hospital.

Sf9 was obtained from Expression Systems (94-001F); Hi5 (B85502) and ExpiSf9 (A35243) cells were obtained from Thermo Fisher.

| | |
|---|---|
| Authentication | All commercial cell lines were authenticated by Short Tandem Repeat profiling (Genetica). |
| Mycoplasma contamination | All cell lines tested negative for mycoplasma (Sigma-Aldrich). |
| Commonly misidentified lines (See ICLAC register) | No commonly misidentified cell lines were used. |

## Animals and other research organisms

Policy information about studies involving animals; ARRIVE guidelines recommended for reporting animal research, and Sex and Gender in Research

| | |
|---|---|
| Laboratory animals | NOD.Cg-Prkdcscid Il2rgtm1Wjl/SzJ mice (NSG/JAX stock 005557) were implanted with PDX lines at 10+ weeks of age. Mice were housed with a 12-hour light/dark cycle set with lights on from 6 AM to 6 PM, with room temperature kept between 21–23°C, and humidity between 30–80%. Temperature and humidity were continuously controlled and monitored. |
| Wild animals | Wild animals were not used in this study. |
| Reporting on sex | Only female mice were used in the study according to established lab protocol and because experiments only involved transplantation of PDX models into mice for subsequent cell harvesting and ex vivo cellular experiments. Hence sex- and gender-based analyses are not relevant this study. |
| Field-collected samples | Field-collected samples were not used in this study. |
| Ethics oversight | St. Jude Children's Research Hospital Institutional Animal Care and Use Committee approved protocol 589-100536-04/18 |

Note that full information on the approval of the study protocol must also be provided in the manuscript.

## Flow Cytometry

### Plots

Confirm that:

☒ The axis labels state the marker and fluorochrome used (e.g. CD4-FITC).

☒ The axis scales are clearly visible. Include numbers along axes only for bottom left plot of group (a 'group' is an analysis of identical markers).

☒ All plots are contour plots with outliers or pseudocolor plots.

☒ A numerical value for number of cells or percentage (with statistics) is provided.

### Methodology

| | |
|---|---|
| Sample preparation | All flow cytometry was performed on cultured cell lines. For analysis, K562 cells were resuspended, supplemented with Helix NP NIR viability dye, and then measured directly. Live cells were washed with PBS. For FACS sorting, cells were resuspended in cold PBS with 5% fetal bovine serum and Helix NP NIR viability dye, followed by passage through a cell strainer. |
| Instrument | Cell cycle data acquisition was performed on ACEA NovoCyte flow cytometer using NovoExpress software (version 1.6.1). Cell sorting was performed on a MoFlo Astrios Cell Sorter (Beckman Coulter). |
| Software | All flow cytometry data were analyzed using NovoExpress Software (v1.6.1). |
| Cell population abundance | A minimum of 20,000 cells were acquired for analysis. The relevant cell populations after FACS sorting were analyzed by follow-up flow cytometry where possible (base editor screen cells), confirming high purity. |
| Gating strategy | For all experiments, we began by (1) gating out debris using SSC-H vs. FSC-H, and (2) gating for single cells using FSC-A vs. FSC-H. Additional gating was based on parent control cells assayed in parallel. For fluorescent reporter experiments, we gated for mCherry+ cells such that 99% of non-transduced cells were mCherry-, and subsequently gated for GFP+ cells such that 99% of non-transduced cells were GFP-. For degradation assays, we gated for GFP+ cells such that 99% of untreated cells were GFP+.<br>For knockdown/overexpression experiments, we gated for mCherry+ cells such that 99% of non-transduced cells were mCherry-, and subsequently gated for GFP+ cells such that 99% of non-transduced cells were GFP+.<br><br>Representative gating strategies are presented in Extended Data |

☒ Tick this box to confirm that a figure exemplifying the gating strategy is provided in the Supplementary Information.
